# Gold Nanosystems Covered with Doxorubicin/DNA Complexes: A Therapeutic Target for Prostate and Liver Cancer

**DOI:** 10.3390/ijms232415575

**Published:** 2022-12-08

**Authors:** Rosa M. Giráldez-Pérez, Elia Grueso, Antonio J. Montero-Hidalgo, Raúl M. Luque, José M. Carnerero, Edyta Kuliszewska, Rafael Prado-Gotor

**Affiliations:** 1 Department of Cell Biology, Physiology and Immunology, Faculty of Sciences, University of Cordoba, 14014 Cordoba, Spain; 2 Department of Physical Chemistry, Faculty of Chemistry, University of Seville, 41012 Seville, Spain; 3 Maimonides Biomedical Research Institute of Cordoba (IMIBIC), Reina Sofia University Hospital (HURS), Department of Cell Biology, Physiology and Immunology, University of Cordoba, 14004 Cordoba, Spain; 4 CIBER Physiopathology of Obesity and Nutrition (CIBERobn), 14004 Cordoba, Spain; 5 Chemtra, 47-300 Krapkowize, Poland

**Keywords:** chemotherapy, gold nanoparticles, DNA compaction, gemini surfactants, doxorubicin

## Abstract

Different gold nanosystems covered with DNA and doxorubicin (Doxo) were designed and synthesized for cancer therapy, starting from Au@16-Ph-16 cationic nanoparticles and DNA–Doxo complexes prepared under saturation conditions. For the preparation of stable, biocompatible, and small-sized compacted Au@16-Ph-16/DNA–Doxo nanotransporters, the conditions for the DNA–Doxo compaction process induced by gold nanoparticles were first explored using fluorescence spectroscopy, circular dichroism and atomic force microscopy techniques. The reverse process, which is fundamental for Doxo liberation at the site of action, was found to occur at higher C_Au@16-Ph-16_ concentrations using these techniques. Zeta potential, dynamic light scattering and UV–visible spectroscopy reveal that the prepared compacted nanosystems are stable, highly charged and of adequate size for the effective delivery of Doxo to the cell. This fact is verified by in vitro biocompatibility and internalization studies using two prostate cancer-derived cell lines (LNCaP and DU145) and one hepatocellular carcinoma-derived cell line (SNU-387), as well as a non-tumor prostate (PNT2) cell line and a non-hepatocarcinoma hepatoblastoma cell line (Hep-G2) model used as a control in liver cells. However, the most outstanding results of this work are derived from the use of the C_I_+N_I_ combined treatments which present strong action in cancer-derived cell lines, while a protective effect is observed in non-tumor cell lines. Hence, novel therapeutic targets based on gold nanoparticles denote high selectivity compared to conventional treatment based on free Doxo at the same concentration. The results obtained show the viability of both the proposed methodology for internalization of compacted nanocomplexes inside the cell and the effectiveness of the possible treatment and minimization of side effects in prostate and liver cancer.

## 1. Introduction

Cancer is the second leading cause of death worldwide and is responsible for around 10 million deaths per year; approximately, 1 in 6 deaths are due to cancer [1]. According to the International Agency for Research on Cancer in 2020, age-adjusted incidence rates of prostate cancer are between 30 and 84 years. On the other hand, liver cancer causes 30.7% of deaths in men between the ages of 30 and 84, and 11.6% in women between 30 and 84 years of age [2].

Doxo is an anthracycline antibiotic, isolated from the species *Streptomyces peucetius* and used effectively in a variety of cancers; it usually inhibits DNA polymerase, thereby inhibiting DNA synthesis. It also inhibits RNA synthesis and its transcription through RNA polymerase inhibition [3,4]. Furthermore, cellular damage has been described at the mitochondria level due to the proliferation of reactive oxygen species (ROS) [5]. These effects on mitochondrial metabolism are very extensive [6]. It has a broad spectrum of use, in the treatment of cancers in adults and children, including solid tumors and hematological malignancies [7,8]. In addition to being used for liver tumors and prostate cancer, Doxo is also used to treat acute leukemia, breast cancer and childhood solid tumors and non-Hodgkin’s lymphomas, as well as Hodgkin’s and soft tissue sarcomas [9,10,11,12,13,14]. Despite being highly effective, Doxo is not selective for cancer cells, which significantly limits its use due to toxicity problems. This toxicity often affects the heart, brain, liver, and kidneys, and the consequences of these toxicities can take many years to become apparent [15]. It would, therefore, be very useful to find mechanisms for the administration of this anticancer compound, which would be conducive to a more direct action on malignant cells while protecting healthy cells.

A good model for this purpose could be the use of small nanosystems as vehicles to transport this anticancer drug, alleviating the problems associated with dosage, biocompatibility and toxicity. In fact, nanoscale drug delivery systems with particle diameters up to 100 nm seek to overcome some of the limitations of traditional anticancer drug therapy [16]. One such system is used to transport lipids [16]. In fact, the liposomal injection of Doxo was the first nanoscale delivery system approved in clinical therapy, specifically in the treatment of patients with Kaposi’s sarcoma associated with acquired immunodeficiency syndrome [17]. These drug-carrying liposomes are used to decrease drug toxicity and to increase antitumor efficacy. However, in the majority of studies performed with liposome-associated anticancer drugs, those that are less toxic than non-encapsulated formulations show some specific adverse effects of liposomes, such as various skin reactions and hypersensitivity reactions [18,19]. For example, hypersensitivity reactions were experienced in ovarian cancer patients treated with liposome-associated Doxo during their first cycle of chemotherapy [19]. In other types of treatments, patients experience different hypersensitivities ranging from hypotension or hypertension, dyspnea, flushing and skin rash to a sensation of suffocation [20,21,22,23,24,25,26,27,28]. Other studies show how Doxo treatment induces lipid peroxidation and suggest that it is a contributing factor to Doxo cardiotoxicity [29,30].

Due to the prevalence of problems related to toxicity, hypersensitivity reactions and cardiotoxicity in liposome-associated formulations, the use of nanosystems as new vectors for cancer chemotherapy is gaining relevance as a possible alternative in recent years. For instance, functionalized selenium nanoparticles with the capacity to bind Doxo were tested on HepG2 liver tumor cells [31]. These nanoparticles were able to bind siRNA (anti-Nanog) by electrostatic interaction, inducing the compaction of the biopolymer and giving complexes of 12 nm mean size [31]. In another study, 20–25 nm core sized AuNPs (gold nanoparticles) modified with polymers, such as poly(ethylene glycol), and associated with carboxylated PAMAM G4 dendrimers were synthesized [32]. This system was tested in lung cells improving the intracellular release of Doxo in vitro through the enhanced permeability and retention effect [32]. Another example shows the synthesis and internalization in MCF-7 breast cancer cells of mesoporous silica nanoparticles wrapped in graphene oxide (MSN@GO), with a variable size from 70 to 80 nm, for the joint delivery of cinnamaldehyde and Doxo [33]. Therefore, drug delivery systems based on biopolymers and nanoparticles have been intensively studied in recent years. In this regard, new strategies employed biopolymer-based nanosystems for doxorubicin targeted delivery with a hydrodynamic size of 80–150 nm. These nanosystems used poly-γ-glutamic acid (PGA) and chitosan (CH) as biodegradable polymers, and they are able to deliver greater tumor growth inhibition, with less general toxicity than the free Doxo [34]. In this context, another strategy based on the use of magnetoliposomes loaded with doxorubicin produced nanosystems able to effectively and selectively reduce the viability of human breast tumor cell lines [35]. Other similar strategies that employ DNA as a biopolymer used Doxo-loaded DNA nanoparticles for the treatment of ovarian cancer, where results from immunohistochemical analysis demonstrated low number of proliferative cells in the ovarian tumor tissue [36]. As can be demonstrated, the coating of the system with a biopolymer such as DNA leads to an improvement in the drugs’ biocompatibility, providing colloidal stability and improving the systemic circulation time [37,38,39,40].

In this work, the great versatility of AuNPs as vehicles/targets for the transport/ release of Doxo to prostate and liver cancer cells is highlighted employing AuNPs of small mean diameter [41], which contributes to their internalization in cancer cells. This novel strategy is based on the transport of the anticancer drug using DNA compacted gold nanocomplexes as a vehicle.

However, the transport of medicines linked to large DNA biopolymers to the interior of the cell requires the fulfillment of at least two fundamental characteristics: high efficiency in transfection and low toxicity [42,43,44,45]. Besides, the correct efficiency in the transport of the DNA–drug-type nanocomplexes to the cell’s interior requires the following. (i) Minimization of the electrostatic repulsion existing between the highly negatively charged phosphate groups of DNA and the cell surface, and the cell surface, of identical charge. This can be achieved by complexing DNA with cationic agents capable of neutralizing the charge of the outer double strand, e.g., AuNPs covered with cationic gemini surfactants, such as Au@16-Ph-16. (ii) Overcoming the steric restrictions implied by the transfer of large polymers within the cell membrane. This specific restriction can be resolved with the use of an external compacting agent, in which Au@16-Ph-16 precursors play a key role in DNA–Doxo complex compaction. Despite the fact that a great number of systems are able to induce DNA compaction [46,47,48], the number of reagents capable of inducing DNA decompaction is much smaller [44]. The decompaction phenomenon of the polynucleotide is necessary for the effective release of the drug inside the cell membrane. In this sense, the means by which cationic gemini surfactants are able to induce effective and reversible DNA compaction depending on the surfactant/biopolymer concentration ratio (C_surfactant_/C_DNA_) is relevant, as has been demonstrated in numerous studies [39,49,50]. For instance, in the absence of any added nanoparticles, N,N’-[1,3-phenylenebis(methylene))bis[N,N-dimethyl-N-(1-hexadecyl)]-ammonium dibromide (16-Ph-16) gemini surfactant is able to induce DNA compaction at low C_surfactant_/C_DNA_ = 0.6 (and low 16-Ph-16 concentrations, C_16-Ph-16_) and subsequent decompaction at high R = 10 (and high C_16-Ph-16_) [39]. In an attempt to apply this particular advantage to the transport/release of Doxo to cancer cells mediated by gold nanoparticles, we have designed and synthesized AuNPs covered with the 16-Ph-16 surfactant, which are then covered with DNA–Doxo complexes. In this configuration, DNA biopolymer is used both as a glue that holds the integrity of the nanostructure and for improving biocompatibility. Thus, the strong interaction between the 16-Ph-16 cationic surfactant linked to the gold and the DNA which transport the Doxo drug is mediated by the partial intercalation of the surfactant between the DNA base pairs (K_16-Ph-16/DNA_ = (8.8 ± 1.8) × 104 (M)), guaranteeing the formation of stable Au@16-Ph-16/DNA–Doxo complexes [39]. The design and preparation of the nanomedicine entailed two consecutive steps. First, it allowed the incorporation of cationic gemini surfactants of the nanoparticle to anionic DNA–Doxo complexes via fundamentally electrostatic and hydrophobic interactions. Second, the appropriate Au@16-Ph-16/DNA molar ratios for inducing both DNA–Doxo compaction/decompaction processes were explored. To do this, different spectroscopic, structural and characterization techniques were employed, adapting the experience acquired in previous studies to the new nanocomplexes [38]. Once the optimal working conditions were established, we proceeded to study both the cell viability and the internalization of the compacted complexes Au@16-Ph-16/DNA–Doxo (C_I_) in different prostate and liver cell lines, as well as the effect that the addition of nanoparticles at the appropriate concentration (N_I_) had on the decompaction and the release of the drug inside the cell. The results obtained show the feasibility of the proposed methodology in both the internalization of compacted nanocomplexes inside the cell and the effectiveness of the treatment against cancer cells.

Furthermore, in this research, we have shown for the first time that, at the cellular level, with the results obtained in both the TEM and viability studies, the addition of free N_I_ induces decompaction of the Cs nanocomplexes contained by the anticancer agents. The aim of this work is to obtain nanosystems of Doxo transporters capable of selectively attacking cancer cells, avoiding possible side effects. In this regard, two nanosystems play a key role in this strategy: (i) the compacted nanosystems C_I_, which serve as nanocarrier for DNA/Doxo complexes, and (ii) the cationic gold nanoparticles N_I_, which act as decompacting agent for DNA/Doxo complexes delivering the drug inside the cells. In order to select the better nanosystem to avoid possible side effect in normal cells, different nanoformulations were explored varying their nanoparticle composition and were then tested using tumorigenic and non-tumorigenic control cell lines that are well established in the literature. As a result, the combined effect that C_I_ + N_I_ nanosystems exert was noteworthy for specific nanoformulations due to the different therapeutic effect that they exert against cancer and normal control cells. This effect is especially relevant in cancer cells in the case of the C_3_ + N_3_ nanosystem because it increases the effect of the drug with respect to free Doxo, thereby enhancing the anticancer effect in the short term (24–48 h). However, this combined nanosystem exerts a protective effect in normal cells with respect to the toxicity of free Doxo. Importantly, this last effect is more marked, especially in the case of more aggressive tumor cells of both liver and prostate cancer cells. That is, the application of C_3_ + N_3_ treatment resulted in viabilities of 39% and 11% for SNU387 and DU145 cancer lines, respectively, while the free Doxo reported viabilities higher than 60% in the same experimental conditions. Therefore, the selectivity of the new treatment towards cancer cells makes it possible to minimize the side effects derived from systemic treatments due to the administration of free anticancer agents.

## 2. Results and Discussion

### 2.1. Conformational Changes in DNA/Doxo Complexes Induced by Au@16-Ph-16 Cationic Nanoparticles: Au@16-Ph-16/DNA–Doxo Complex Formation

**Figure 1:**
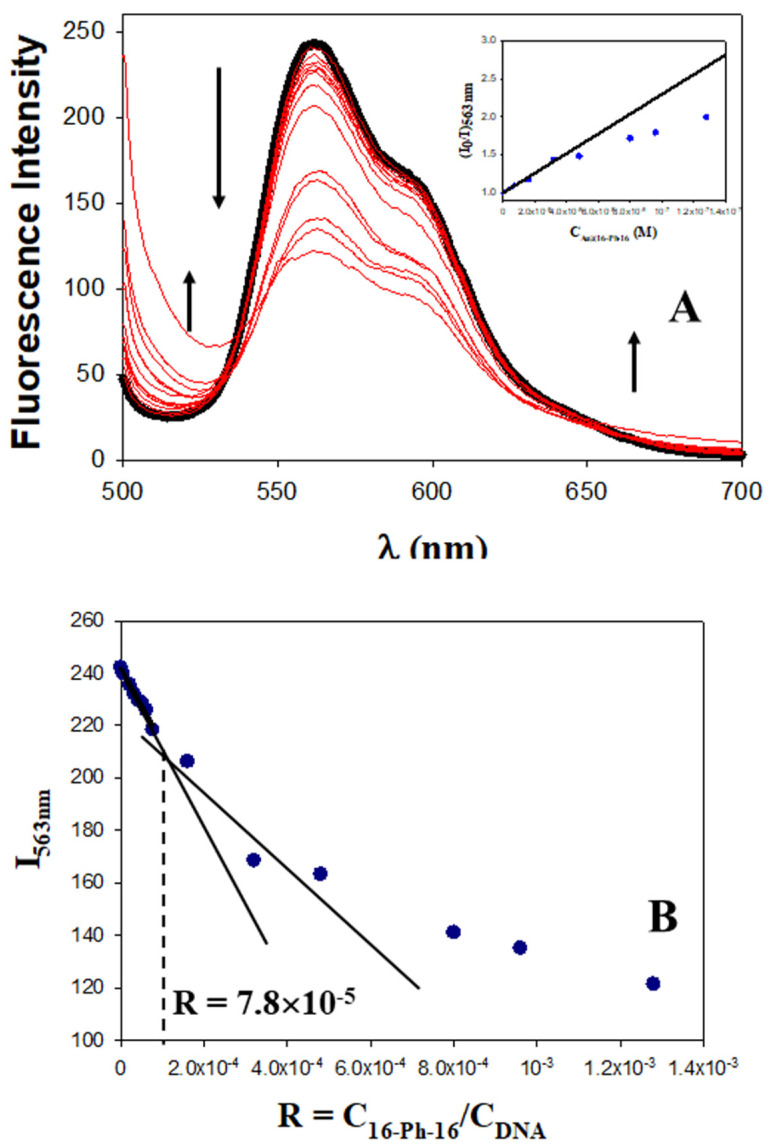
Fluorescence experiments with the DNA/Doxo system in the presence of different C_Au@16-Ph-16_, at 298.0 K in cacodylate buffer (I = 1.63 mM, pH = 7.4); C_Doxo_ = 2.5 μM and C_DNA_ = 100.0 μM remain constant. (**A**) Fluorescence spectra of the DNA/Doxo system in the absence and presence of Au@16-Ph-16 nanoparticles. C_AuNPs_ = 0 M (in black), C_AuNPs_ = 0.0053–128 nM; the arrow indicates change (in blue). The inset shows the Stern–Volmer plot of the DNA/Doxo system at different C_Au@16-pH-16_. Deviation from the linearity occurs at C_Au@16-Ph-16_ > 3.0 × 10^−8^ M. (**B**) Plot of I_563 nm_ versus the molar ratio, R = C_Au@16-Ph-16_/C_DNA_.

Characterization of nanoparticles with different physicochemical techniques is very useful to predict clinical efficacy. In fact, as is known, various key physicochemical properties of nanomaterials have a synergistic effect on their cellular entry pathways [51]. Thus, the physical barrier for nanomedicine uptake is determined by the physical properties of both nanoparticles and membranes, where nanoparticle size, shape, core rigidity, membrane thickness and stiffness constitute key parameters. For instance, in the case of the endocytosis translocation process, both nanoparticle size and membrane thickness constitute physical barriers [51]. Furthermore, in order to obtain clinically effective products and diminish the time and effort needed to complete the transition from benchtop to point of care, adequate methodologies are imperative to characterize nanomedicines and correlate their effects, which take into account physicochemical characterization. As is known, nanoparticle concentration has an important effect on physicochemical properties, such as surface charge, size, composition and aggregation state of the nanoformulations [51,52,53]. Moreover, in general, it has been observed that toxicity decreases with higher concentrations of nanoparticles [54]. For all these reasons, we decided to explore distinct nanoformulations with varying reagent concentrations. Monitoring the changes in the fluorescence of ligand–receptor systems with the ligand/receptor molar ratio is one of the simplest and most appropriate methodologies to detect complex formation. In the present study, we first prepared DNA/Doxo complexes under saturation conditions and then studied the changes in the fluorescence emission of the DNA/Doxo system caused by varying C_Au@16-Ph-16_ content, and consequently the R = C_Au@16-Ph-16_/C_DNA_ molar ratio (see Figure 1).

Fluorescence spectra in Figure 1 corresponding to the Au@16-Ph-16/DNA–Doxo complex show two not well-defined inflection points at about 540 nm and 650 nm, which indicates that the binding is probably not a simple process. However, according to Hamilton and Naqvi, the appearance of an isosbestic point in fluorescence spectroscopy must be examined carefully, due to the possible influence of the excited state [55]. Moreover, between the two inflection points, the fluorescence of the DNA/Doxo system decreases significantly with C_Au@16-Ph-16_, while it increases below 540 nm and above 650 nm, which is indicative of the Au@16-Ph-16/DNA–Doxo complex formation (see Figure 1A). Note that in these experiments, C_DNA_ and C_Doxo_ remain constant. Thus, C_Au@16-Ph-16_ changes in the same way as the R ratio. Moreover, a closer examination of Figure 1B shows two different behaviors in the system as C_Au@16-Ph-16_ and R molar ratio increase: (i) a smoother descent in the fluorescence at 563 nm at low C_Au@16-Ph-16_ that can be assigned to the formation of Au@16-Ph-16/DNA–Doxo complexes, and (ii) a more pronounced decrease in the intensity at 563 nm at the high C_Au@16-Ph-16_ probably related to the existence of a conformational change in the biomolecule starting at R = 7.8 × 10^−5^.

The quenching efficiency of gold nanoparticles corresponding to the first descent in the fluorescence emission of the DNA/Doxo complexes, was evaluated in Figure 1A (see the inset) using the Stern–Volmer plot (I_0_/I = 1 + K_SV_ × C_Au@16−Ph−16_), where I_0_ and I are the fluorescence intensities in the absence and presence of Au@16-Ph-16, respectively, and K_SV_ is the Stern–Volmer constant. A K_SV_ value of (1.29 ± 0.09) × 10^7^ M^−1^ was obtained, confirming the notable interaction between gold nanoparticles and DNA/Doxo complexes. Deviation from linearity was observed from C_Au@16-Ph-16_ = 0.32 nM and R = 7.8 × 10^−5^, coincident with the R ratio at which a possible conformational change is induced in the biomolecule.

**Figure 2:**
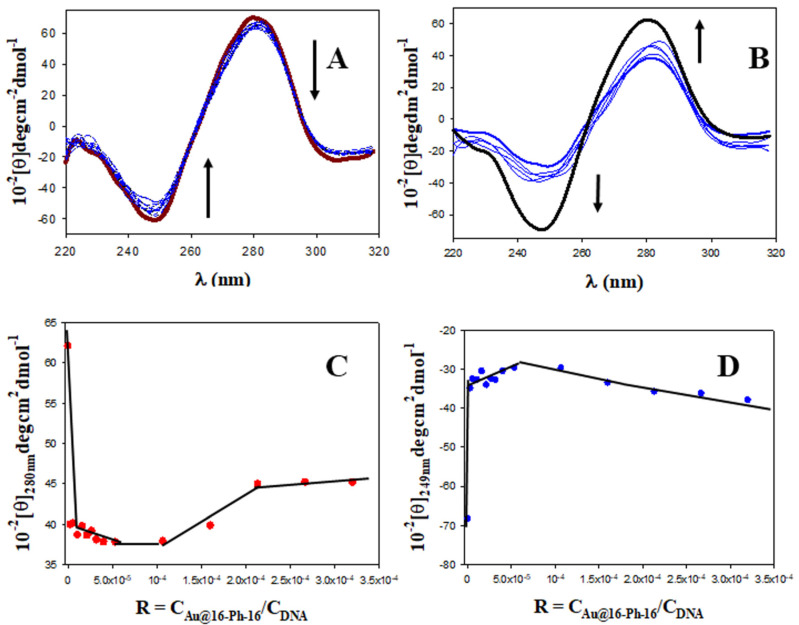
CD titrations of the DNA–Doxo system at different C_Au@16-Ph-16_ in cacodylate buffer (I = 1.63 mM, pH = 7.4); C_Doxo_ = 5 μM and C_DNA_ = 200.0 μM. CD spectra of free DNA and DNA/Doxo complex in black and red, respectively. (**A**) First behavior; the arrow indicates change at 0, 0.0053, 1.07, 2.13, 3.20, 4.27, 5.33, 6.40, 8.00, 10.7 and 21.3 nM of Au@16-Ph-16. (**B**) Second behavior; the arrow indicates change at 21.3, 32.0, 42.7, 53.3 and 64.0 nM of Au@16-Ph-16. (**C**,**D**) Plots of [θ]_280 nm_ and [θ]_249 nm_ versus R.

To confirm or disprove the hypothesis described, it is necessary to employ complementary spectroscopic and structural techniques. Therefore, in order to explore the possible conformational changes that occur upon binding of Au@16-Ph-16 to the DNA/Doxo complex, CD spectra were performed in the absence and in the presence of gold nanoparticles. Figure 2A (in black) shows a characteristic spectrum of the right-handed B-form of DNA in extended conformation in the intrinsic CD region (220–320 nm), which has a positive peak at about 280 nm and a negative peak at approximately 249 nm. Note that these CD bands are caused by both the stacking interactions between the DNA bases and by the helical suprastructure of the polynucleotide that provides an asymmetric environment for the bases [56]. Structural alterations in the DNA biomolecule caused by interactions with different ligands result in changes in this far-UV region [57,58]. The same figure shows that the CD changes induced by the addition of Doxo (Figure 2A, in red) lead to remarkable perturbations in both positive and negative bands, demonstrating that the helical conformation is not maintained upon binding. These conformational changes, consisting of an increase in both the negative and positive CD bands, without an appreciable shift in their position, are compatible with Doxo intercalation within DNA base pairs [59]. However, when gold nanoparticles were added to the intercalated DNA/Doxo complex, the observed CD behavior was completely different. That is, a decrease in the intensity of both CD bands was accompanied by a small shift in the positive CD band to higher wavelengths (Figure 2A, in blue), indicating the compaction of the DNA complex and partial denaturation of the double strand [49,50,60], and, therefore, DNA/Doxo compaction.

Note that DNA/Doxo compaction and binding with Au@16-Ph-16 can be expected to occur principally through favorable electrostatic interaction between the negative phosphate groups of the DNA complex and the positive charge of the gold nanoparticles [40], as well as through hydrophobic interaction between the surfactant tail and the DNA bases. Subsequently, it is noteworthy that this tendency to DNA compaction is reverted at higher C_Au@16-Ph-16_ and R ratios (see Figure 2B). At about R = 8·10^−5^ (see Figure 2C,D), inflection points in the plot of [θ]_280 nm_ and [θ]_249 nm_ versus R are observed, after which the intensities of the negative and positive CD bands increase until values below those presented by free DNA in solution are reached (Figure 2B, in black). This behavior indicates a partial decompaction of the polynucleotide induced at a high ratio of concentrations. However, the nature of the conformational changes observed must be confirmed using the ultrasensitive AFM technique.

**Figure 3:**
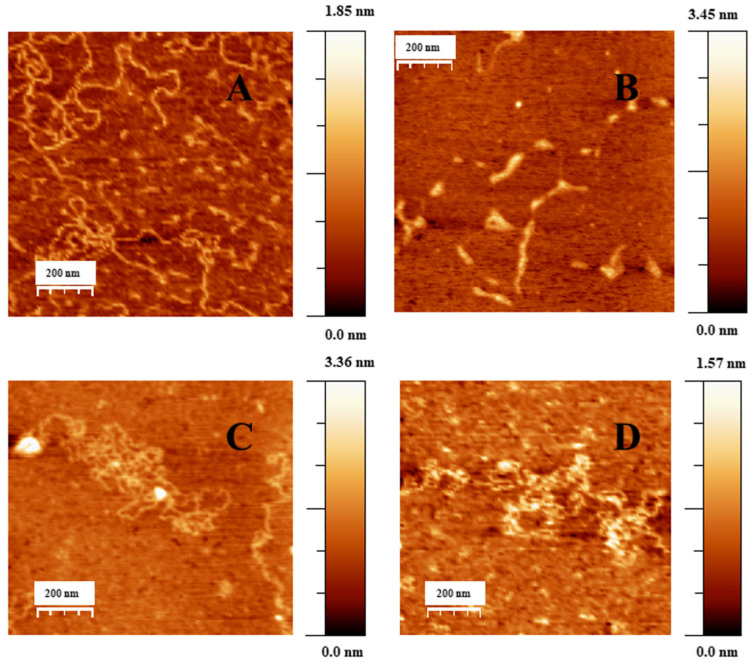
AFM topography image of DNA and DNA/Doxo complexes adsorbed on the APTES modified mica surface in cacodylate buffer (I = 1.63 mM, pH = 7.4), C_DNA_ = 0.3 μM. (**A**) DNA in extended-coil conformation in the absence of Doxo. (**B**–**D**) Structure of DNA–Doxo complexes, C_Doxo_ = 7.5 nM.

Figure 3A shows an AFM topographic image of free double stranded DNA adsorbed onto the APTES modified mica surface in extended coil conformation. Figure 3B–D shows the formation of different DNA–Doxo complexes, in which multiple intramolecular and intermolecular loops are formed. Moreover, some random parts of the DNA chains are condensed, and some crossover points are visualized.

**Figure 4:**
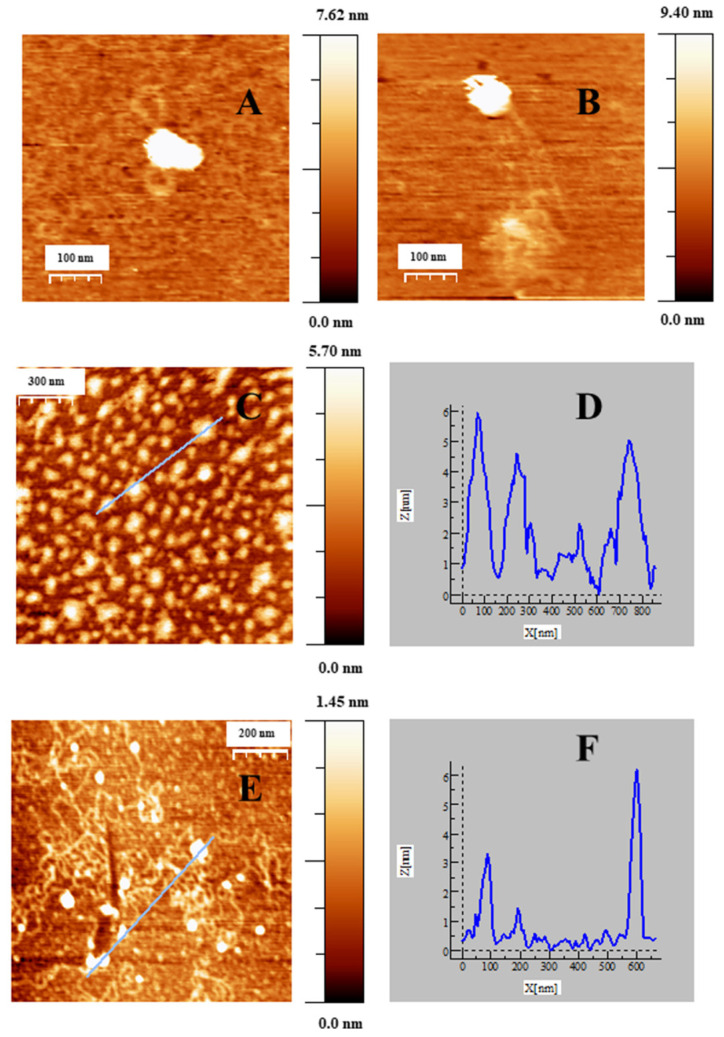
AFM topography images of C_I_ complexes adsorbed on APTES modified mica surface in cacodylate buffer (I = 1.63 mM, pH = 7.4; C_DNA_ = 0.3 μM, C_Doxo_ = 7.5 nM), under different reaction conditions. (**A**,**B**) Intermediates of compaction, C_Au@16-Ph-16_ =1.07 pM; (**C**) C_I_ complexes at a compact stage, C_Au@16-Ph-16_ = 22.5 pM. (**E**) C_I_ complexes at a decompacted stage, C_Au@16-Ph-16_ = 124.5 pM. (**D**–**F**) correspond to the cross section along the selected lines for (**C**,**E**).

When low C_Au@16-Ph-16_ was added to the DNA–Doxo complexes, we observed the progressive compaction of the system (see Figure 4A–D). At low R = 3.6 × 10^−6^, Figure 4A,B show the formation of intermediates in the DNA compaction process induced by gold nanoparticles. Thus, the association among the hydrophobic chains of 16-Ph-16 in gold nanoparticles with the DNA biopolymer in the DNA–Doxo complex, together with the favorable electrostatic interaction between the negative phosphate groups of DNA and the cationic polar head of the gemini surfactants, promotes the formation of intramolecular DNA condensates. DNA loops and extended DNA chains emerging from compact globules can both be seen in these structures. Note that similar structures were observed with cationic monomeric and dimeric surfactants CTAB and 12–3–12 with DNA, in which reversible DNA compaction was accomplished at low surfactant–DNA concentration molar ratios [49,61]. Subsequently, when more C_Au@16-Ph-16_ is added to the complex (R = 7.5·10^−6^), see Figure 4C–D, globular compact structures with an average diameter of 67 ± 18 nm and a height of 3.7 ± 0.9 nm can be observed. It is noteworthy that smaller globules were obtained in this approximation in comparison with previous cationic surfactant bromide of cetyltrimethylammonium (CTAB) and gemini surfactant 12–3–12 systems in the absence of gold nanoparticles. This finding clearly shows the potential of AuNPs in DNA compaction [49,61]. Note that the R ratio at which the completion of the compaction process is reached is in good agreement with previously described CD and fluorescence results, in which an inflection point or a change in DNA conformation was observed at about R ~ 8 × 10^−6^. Again, the effectiveness of the new synthesized system in DNA compaction is clear compared with similar systems in the absence of nanoparticles. That is, a concentration ratio of 0.5–0.6 was needed for the DNA–CTAB system and 0.25 for the 12–3-12 analogous system. In the present study, a smaller quantity of gold nanoparticles was needed to obtain similar results, which demonstrates the advantage of using gemini surfactant-covered gold nanoparticles from an economic point of view, as well as for the transport of the drug to the cell and subsequent effective release driven by biopolymer decompaction. Note that the effect of gold nanoparticles covered with 16-Ph-16 gemini surfactants is again noteworthy compared with the analogous DNA–16-Ph-16 system in the absence of any added gold [39,62], in which a surfactant–DNA concentration ratio of 5–10 was needed for the decompaction of the biomolecule, demonstrating the advantage of the new design based on gold nanoparticles. Finally, when the morphology of DNA structures at higher C_Au@16-Ph-16_ and R ratio was explored, it was observed that the Au@16-Ph-16/DNA–Doxo compacted complexes formed at low R undergo a change in conformation, which is compatible with an increase in the ellipticity of both positive and negative CD bands (see Figure 2B). That is, Figure 4E,F shows that the DNA experiences a partial decompaction process at R = 4.2 × 10^−4^, which is again in agreement with the fluorescence and CD results. The identification of the R ratio condition for compaction/decompaction processes in DNA–Doxo complexes induced by low or high C_Au@16-Ph-16_ is a key factor for the correct design and synthesis of Au@16-Ph-16/DNA–Doxo complexes for the transport of Doxo to the cell and the subsequent effective release of the drug driven by biopolymer decompaction.

### 2.2. Charge, Size and Stability of Au@16-Ph-16 and Au@16-Ph-16/DNA–Doxo Nanosystems

Particle size, the most basic information about nanoparticles, is one of the main determinants of biodistribution and the retention of nanoparticles in target tissues [63]. In this sense, nanosystem size is a parameter that plays a key role in the enhancement of the permeability and retention effect of anticancer drugs in tumors. For instance, nanosystems in the size range of 50–200 nm can extravasate and accumulate effectively inside the tumor tissue and inflammatory sites, providing therapeutic benefits [64,65]. Moreover, the charge properties determined by the zeta potential parameter critically influence not only the interaction processes of a nanosystem with the environment, but also clearance processes [52,53]. For instance, taking into account the surface charge, there is a clear correlation between NP charge and the clearance time of the nanomedicine; that is, cationic NPs are generally more rapidly cleared than anionic NPs, followed by neutral and slightly negative NPs, which have the longest half-lives in circulation [53]. Thus, the stability of nanoparticles is controlled by the valence of the counterions in the solution due to the electrokinetic or zeta potential (ζ) corresponding to the difference between the compact layer potential and diffuse potential. Thus, the measured ζ potential for a given nanosystem is an indication of the repulsive force that prevents nanoparticle aggregation, maintaining colloidal gold stability. In this sense, a zeta potential value of around +/−30 mV or higher is considered optimum to attain better physical colloidal stability [66]. Moreover, maintaining nanoparticle stability in the drug delivery process is a key condition to guarantee its correct transport to the target tissues [51]. For example, a high polydispersity index (PDI) indicates low particle homogeneity and eventual loss of special nanoscale properties. In general, their stability is due to the repulsive electrostatic force which nanoparticles experience; thus, selecting the proper stabilizer is crucial for optimizing drug delivery systems [67]. Stable nanosystems fabricated using controlled manipulation of materials and optimal synthesis conditions can serve as drug transporters for carrying the drug in a controlled manner from the site of administration to the target site in the body [68]. In fact, targeted delivery can potentially reduce toxicity and increase the efficacy of drugs, reducing the dosage needed in cancer treatment. However, stability is not only influenced by the selected surface chemistry, but also by different factors such as aggregation state, nanoparticle composition, shape and size, for which zeta potential, DLS and UV–visible techniques are crucial. For instance, a simple broad-spectrum technique used to determine nanoparticle stability over time consists of following the stability of the plasmon resonance signal using the UV–visible technique [69]. Due to all of these reasons, we employed DLS, zeta potential and UV–visible techniques to characterize the synthesized gold nanoparticles and nanocomplexes.

Table 1 shows ζ potential values for different C_I_ and N_I_ nanoformulations. In the absence of the DNA–Doxo complexes, Au@16-Ph-16 nanoparticles at different C_Au@16-Ph-16_ show values in water varying between (+30−+37) mV. This fact, together with the existence of a single zeta potential peak for each N_I_ (see also Figure S5), ensures that the nanoparticles are well separated, away from each other, thus achieving monodispersed and stable nanosystems. Moreover, the ζ potential value of N_I_ formulations indicates that these nanoparticles are positively charged in water media.

As can be seen in Table 1, when the Au@16-Ph-16/DNA–Doxo compacted nanosystems are formed in water, the zeta potential becomes negative, as is to be expected due to the association of DNA–Doxo complexes on the gold surface. Thus, the zeta potential of pure calf thymus DNA measured in water (without nanoparticles or Doxo) was around −60 mV due to the negatively charged phosphate groups on its backbone [70]. As the concentration of the Au@16-Ph-16 increased in the C_I_ nanocomplex (see Section 3.1.4. for more details), the negative charges on the DNA decreased; therefore, the zeta potential increased (became less negative) as C_Au@16-Ph-16_ increased from −49 to −35.5 mV (see Table 1 and Figure S6).

When the charges of the same nanoformulations were measured under buffer conditions, a general decrease in their values was observed. Therefore, a destabilization of the systems occurs driven by the addition of salt (see Table 1 and Figures S7 and S8).

Note that the observed behavior of zeta potentials was consistent with that observed in the size of the complexes. Thus, the value of the size of Au@16-Ph-16 systems in water was (5.6 ± 1.9) nm [40]. However, when different C_I_ compacted nanocomplexes, which incorporate DNA–Doxo compacted complexes to the Au@16-Ph-16 nanoparticles, were prepared from the precursors, a general increase in their size was registered (see Table 2 and Figure S9), being compatible with the x-y diameter measured by the AFM technique (see Figure 4C,D).

Furthermore, when the diameter of C_I_ formulation in water and in buffer conditions was compared, we observed a clear increase in the diameters in the presence of salt (see Table 2 and Figure S10). This is because the partial neutralization of the nanocomplex surface decreases the repulsion forces between them, facilitating the aggregation in line with zeta potential results. Moreover, it is important to note that the effect of adding salt is more evident for the C_1_ formulation, when the C_DNA_ is lower, consistent with the known Debye screening effect. Thus, the length of the screening increased moderately with the low salt content, favoring electrostatic interactions among nanoparticles and favoring nanoparticle aggregation, with the expected size increase [71].

**Figure 5:**
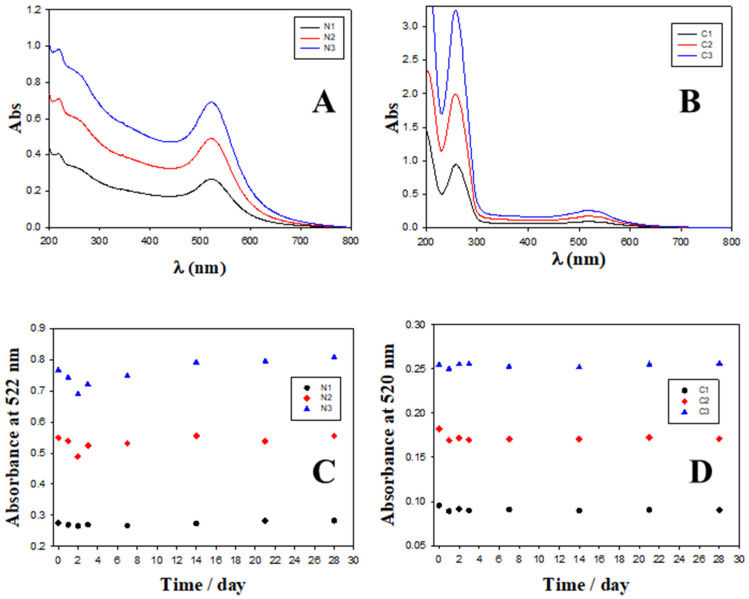
Absorbance spectra and evolution over time of different nanosystems. (**A**) Absorbance spectra of N_1_, N_2_ and N_3_ Au@16-Ph-16 precursors. (**B**) Absorbance spectra of C_1_, C_2_ and C_3_ compacted nanocomplexes. (**C**) Evolution over time of absorbance at fixed 522 nm wavelength (SPR location) for Au@16-Ph-16 precursors at different concentrations; black circles correspond to [Au@16-Ph-16] = N_1_, red diamonds correspond to [Au@16-Ph-16] = N_2_ and blue triangles to N_3_. (**D**) Evolution over time of absorbance at a fixed 520 nm wavelength for compacted nanocomplexes at different concentrations; black circles correspond to [Au@16-Ph-16/DNA–Doxo] = C_1_, red diamonds correspond to [Au@16-Ph-16/DNA–Doxo] = C_2_ and blue triangles to C_3_.

Figure 5A shows the UV–visible plot for the free Au@16-Ph-16 precursor nanoparticle at different C_Au@16-Ph-16_, where the position of the plasmon peak is λspr = 522 nm. Figure 5B corresponds to the UV–visible spectra of C_I_ formulations, in which C_DNA_ and C_Doxo_ were increased from 10 to 28 μM and from 0.25 μM to 0.70 μM, respectively. A slight displacement in the position of the SPR maximum to a lower wavelength (λ_SRR_ = 520 nm, C_I_ formulations) was accompanied by an increase in the absorbance with reference to the corresponding Au@16-Ph-16 precursor in each one (see Figure S2), showing a hyperchromic effect. The SPR behavior of DNA–Doxo complexes after adding Au@16-Ph-16 originates from the difference in morphology of the C_I_ complexes with respect to the corresponding N_I_ precursors. That is, when a DNA biopolymer was incorporated into the precursors, more regular and better dispersed nanosystems with high SPR intensity and sensitivity were obtained in comparison with the non-functionalized Au@16-Ph-16 nanoparticles [72].

On the other hand, the stability of the N_I_ and C_I_ nanoformulations was checked by studying the possible modifications of the complete UV–Vis spectra from 200 to 800 nm over time in situ, at 24 h, 48 h, 72 h, 1 week, 2 weeks, 3 weeks and 1 month after preparation (see Figure 5C–D and Figure S3). Due to the presence of various components in the medium (DNA, Doxo and buffer), the stability of Au@16-Ph-16 nanoparticles can be affected, causing undesired aggregation. Figure 5C–D shows how the intensity of the SPR is practically stable over a month for nanoparticles dispersed in buffer, although appreciable changes were observed in the first few days. In the case of absorbance at 258 nm (see Figure S3A–C) associated with interband transitions (from 200 to 480 nm, approximately) [73], a slight decrease is shown in the first few days and then this value stabilizes. AuNPs are much more reactive in the days after their synthesis, so it is not unusual to observe these variations followed by stabilization. However, when Doxo and DNA were added to the Au@16-Ph-16 colloidal system, the stabilization of the SPR band was observed in 24 h due to their interaction with these biocompounds (see Figure S3D–F). For absorbance at 258 nm, slight oscillations were produced as a reflection of the different modes of interaction of DNA with the colloidal system. Even so, in general, the changes for N_I_ and C_I_ formulations are negligible: the absorbance curves practically superimposed and both the position of the plasmon’s maximum surface and band form were maintained over time for at least 1 month, making it obvious that the system is stable.

On the other hand, the controlled drug release constitutes a key parameter to be improved to ensure the correct clinical translation of new nanomedicine [74]. In this study, the percentage of drug release was evaluated by using the fluorescence spectroscopy technique and the appropriate calibration curve (see Section 3.2.3 for more details and Figures S11 and S12). From such experiments, a %DR of 92.6% and a half life time (t^1/2^) of 11.4 min were obtained. These results clearly show that a high percentage of Doxo was released from the C_3_-compacted nanocomplex after the addition of N_3_ in only 60 min of stabilization (see Figure S12). Therefore, it was demonstrated that the controlled in vitro release of Doxo from the nanocomplex is possible by tuning the DNA conformation from compacted to decompacted forms using the appropriate C_Au@16-pH-16_ concentration, indicating the goodness of the developed nanomedicine.

### 2.3. In Vitro Biocompatibility of Gold Nanosystems

**Figure 6:**
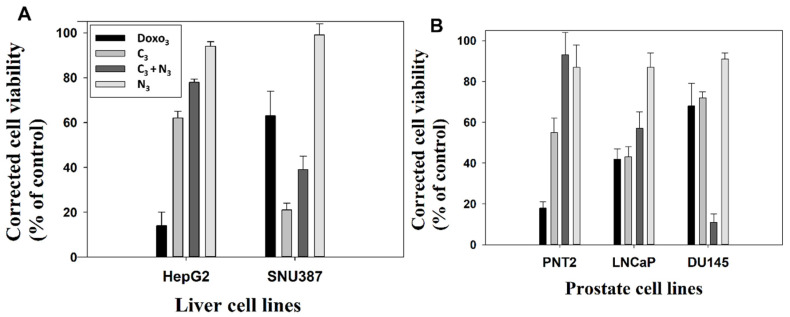
Corrected cell viability of treated cells versus control at 48 h after treatment with Doxo_3_, C_3_, C_3_+N_3_ and N_3_ formulations. Viability of the control corresponds to 100%. (**A**) HepG2 and SNU387 liver cell lines. (**B**) PNT2, LNCap and DU145 cell lines.

Figure 6 and Figures S14 and S15 show the proliferation and viability of the administration of the different treatments. The concentrations of free Doxo, N_I_, C_I_ and C_I_ + N_I_ are the same in each system tested. Different cell lines of liver and prostate cancer were selected to test the effectivity and targeting potential of the developed nanosystems. Specifically, for studying the effect in prostate cancer, we selected the well-known recognized neoplastic cell lines LNCaP and DU145. Grozescu and Popa studied these model systems in their chromosomal composition [75]. PSA secretion by the human neoplastic cells LNCaP is influenced by acute stimuli, such as the Vasoactive Intestinal Peptide (VIP), GHRH (growth hormone-releasing hormone) and chronic stimuli, such as androgens, and have been used for experimentation by numerous authors [75,76,77,78,79,80]. Besides, PNT2 normal prostate epithelial cells were used as a control. This model system has been used as a control in prostate cancer investigations by many authors [78,79,81]. For studying the gold nanosystems effect in liver carcinoma, the well-stablished SNU387 cells and HepG2 were employed as tumorgenic and normal model systems, respectively [82,83,84]. The suitability in the selection of these cell lines for studying liver carcinoma is clearly supported and justified from a high number of investigations. For instance, it was found that the TGF-β1-mediated repression of SLC7A11 drives vulnerability to GPX4 inhibition in hepatocellular carcinoma cells [83]. In another study, these cell lines were used to show how the fibrinogen-like protein 1 modulates sorafenib resistance in human hepatocell carcinoma cells [85]. Studies have also been carried out showing how the expression of the canonical MET transcript is a predictive biomarker of chemosensitivity to MET inhibitors in hepatocellular carcinoma cell lines [84]. Moreover, it is important to note that the genetic and/or pharmacological inhibition of SF3B1 could be verified using these types of cells. Thus, it could constitute a novel and promising therapeutic strategy that is worth exploring through randomized controlled trials [82]. From the present work, we observed that, in general, non-complexed N_I_ nanoparticles were harmless to all types of explored cells. Furthermore, they typically offered a degree of protection when interacting in combination with C_I_ compounds in the particular case of non-tumor liver and prostate cell lines (see Figure 6 and Figures S14 and S15). On the other hand, results derived from the use of C_I_ compacted nanosystems vary depending on the formulation explored. For instance, the results from C_1_ treatment showed significant mortality activity in different tested cell lines, the compacted nanosystem being even more harmful than the corresponding free Doxo_1_ (see Figure S14). Note that in this case, Doxo_1_ serves as reference to measure the effectivity of the new treatment due to it being prepared at the same concentration as in the corresponding C_1_ nanosystem. However, the opposite behavior occurs for the C_3_ formulation in different cell lines with the exception of SNU387 cells. That is, the mortality of cells in the presence of C_3_ was lower than in the case of the corresponding free Doxo_3_, showing a clear protective effect (see Figure 6). Besides, Figure S15 showed that the remaining C_2_ formulation had a variable short-term effect depending on the cell type. Hence, in order to avoid possible side effect in patients, we consider the C_3_ formulation as the more convenient treatment. Importantly, the most striking feature of this work arises from the application of the combined C_I_+N_I_ treatments in different cell lines. That is, we observed a protective effect for all the combined C_I_+N_I_ treatments in all non-tumor cell lines models studied here. However, there was a highly significant destructive effect of the same combined treatment in the explored cancer derived cell lines SNU387, LNCaP and DU145. Moreover, the results showed statistical difference between the cells. As an example of this, the values of the corrected viabilities obtained for C_3_ + N_3_ nanosystems were 39% and 77.9% for SNU387 high-grade hepatocarcinoma and the non-tumorgenic HepG2 model, respectively. The same behavior occurs for prostate cancer lines that showed a viability of 11% for DU145 cell, a model of prostate cancer resistant to conventional treatments, in comparison with the 57% obtained for LNCaP cancer line and the 93% obtained for normal PNT2 line. Hence, the novel combined C_I_+N_I_ treatment is relevant in the sense that it provides a chance for drug combination therapy and inhibition of drug resistance mechanisms using gold nanoparticles. These results can be explained by taking into account the high potential affinity of the gemini surfactant that constitutes the nanoparticles for cancer cells and their capacity for proliferation inhibition [86,87]. As is known, tumor cells have unique pathophysiological features in comparison with non-tumor cells, such as extensive angiogenesis or a faulty vascular network and high energy metabolism [88]. Thus, the results from C_I_ + N_I_ treatments indicate that these nanosystems use the above-mentioned cancer cells features to target tumor tissue more effectively than conventional Doxo treatment, evidencing their high effectivity as anticancer targets

### 2.4. Internalization of Au@16-Ph-16 and Au@16-Ph-16/DNA–Doxo Nanosystems

**Figure 7:**
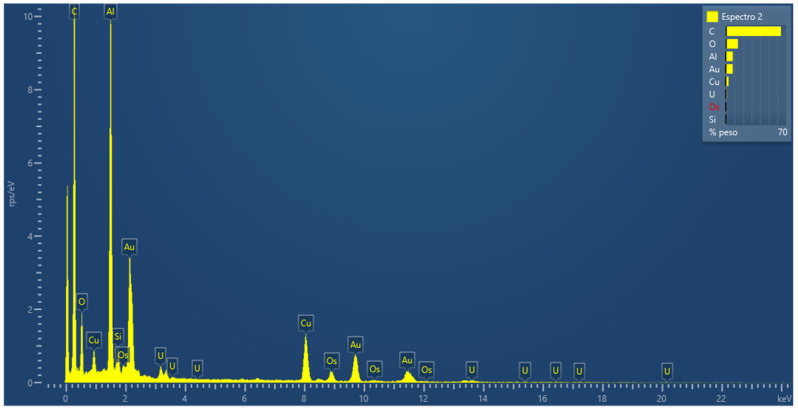
Microanalysis using energy dispersive spectroscopy, in an ultra-fine section of cells fixed, treated and cut in an ultramicrotome, to verify the presence of the gold nanoparticle in the sample.

In parallel to the internalization study, the results of the microanalysis using energy dispersive spectroscopy (EDS) (Figure 7) can verify the different elements resulting from the fixation and contrast treatment and the presence of gold belonging to the nanosystem.

**Figure 8:**
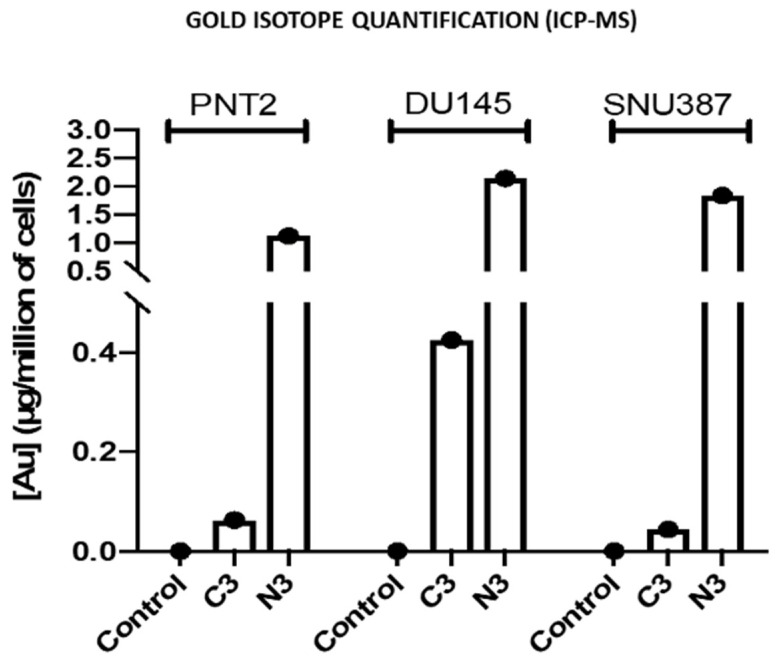
Representation of the concentration of Au isotopes by cell numbers, of the cell lines PNT2, DU145 and SNU 387, used as a validation model by means of an inductively coupled plasma mass spectrometry (ICP-MS) analysis. The data are mean values and the standard deviation (SD), respectively, is SD = 0.03, 0.2 and 0.0018 µg/ million for C_3_, N_3_ and Control in PNT2; SD = 0.04, 0.14, 0.06 µg/million for C_3_, N_3_ and Control in DU145; SD = 0.013, 0.015 and 0.03 µg/million for C_3_, N_3_ and Control in SNU387.

In addition, inductively coupled plasma mass spectrometry (ICP-MS) studies were performed with different samples of the strains studied to check the amount of gold internalized in the cells (Figure 8). The data correlated with the concentrations of gold used in the preparation of the precursors and the nanosystems used, such that as the gold nanoparticle concentration increases in the formulation, a greater concentration of gold is observed by ICP-MS, being higher for the C_3_ and N_3_ formulations. Note that the data for N_3_ are higher than for C_3_. This can be due to N_3_ nanosystems being highly stable, positively charged and smaller in size than C_3_ nanosystems, factors which contribute to a better cellular uptake. In this way, the results obtained with the previous analyses are validated.

**Figure 9:**
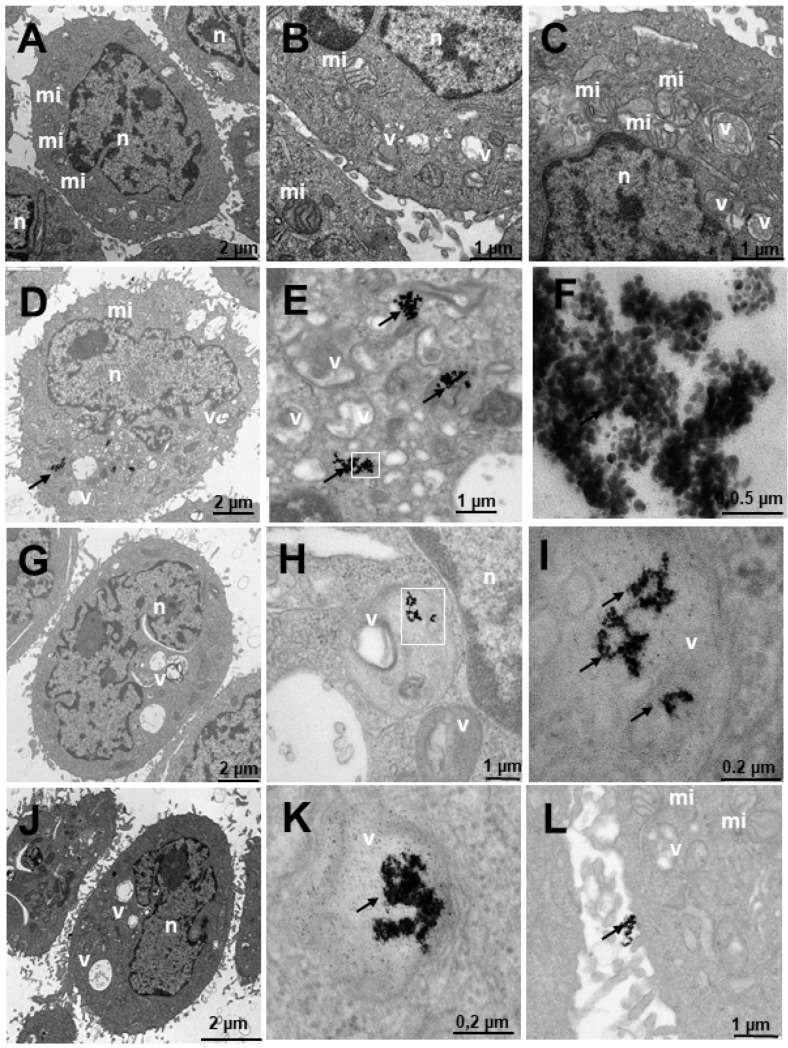
Transmission electron microscopy (TEM) photomicrographs of control cells without treatment (**A**–**C**), compared to Au@16-Ph-16 (N_3_) (**D**–**F**), Au@16-Ph-16/DNA/Doxo (C_3_) (**G**–**I**) and Au@16-Ph-16/DNA/Doxo + Au@16-Ph-16 (C_3_ + N_3_) (**J**–**L**) at 24 h, in non-tumorigenic prostate PNT2 cells. The dense bodies indicated by an arrow are compatible with the gold cores of the nanoparticles. Abbreviations: mi—mitochondria; n—nucleus and v—vesicles.

**Figure 10:**
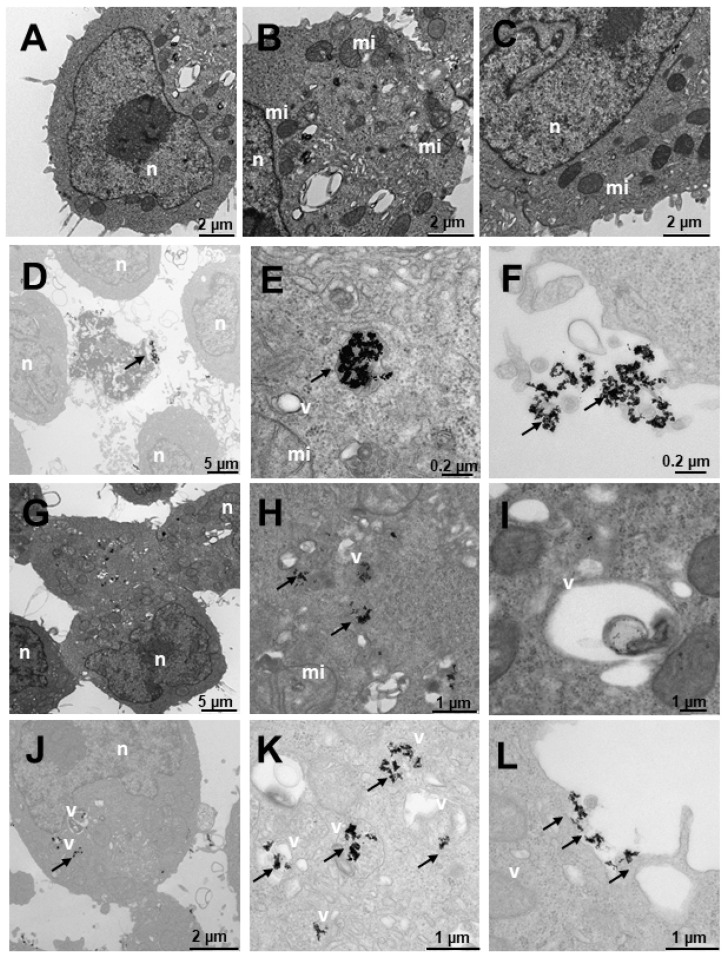
Transmission electron microscopy (TEM) photomicrographs of control cells without treatment (**A**–**C**), compared to Au@16-Ph-16 (N_3_) (**D**–**F**), Au@16-Ph-16/DNA/Doxo (C_3_) (**G**–**I**) and Au@16-Ph-16/DNA/Doxo + Au@16-Ph-16 (C_3_ + N_3_) (**J**–**L**) at 24 h, in LNCaP cells derived from tumorigenic prostate tumor cells from a hormone-sensitive prostate cancer model. The dense bodies indicated by an arrow are compatible with the gold core of the nanoparticles. Abbreviations: mi—mitochondria; n—nucleus and v—vesicles.

**Figure 11:**
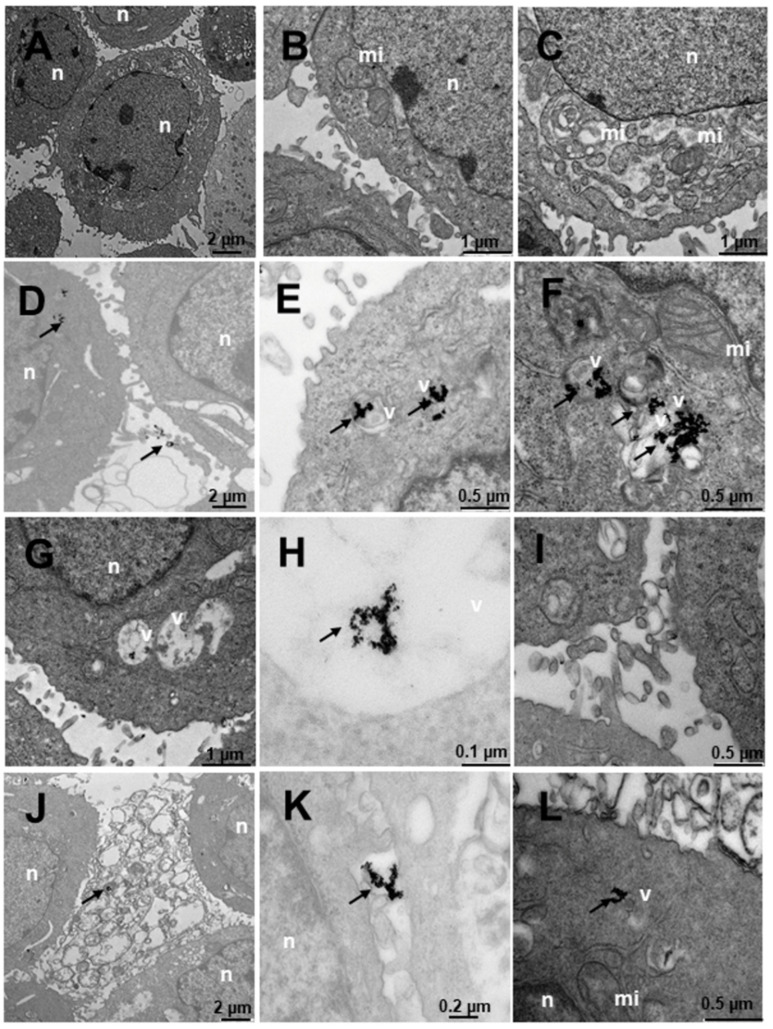
Transmission electron microscopy (TEM) photomicrographs of control cells without treatment (**A**–**C**), compared to Au@16-Ph-16 (N_3_) (**D**–**F**), Au@16-Ph-16/DNA/Doxo (C_3_) (**G**–**I**) and Au@16-Ph-16/DNA/Doxo + Au@16-Ph-16 (C_3_ + N_3_) (**J**–**L**) at 24 h, in DU145 tumor cells derived from prostate tumor, a model of castration-resistant prostate cancer which is not responsive to hormonal therapy. The dense bodies indicated by an arrow are compatible with the gold core of the nanoparticles. Abbreviations: mi—mitochondria; n—nucleus and v—vesicles.

**Figure 12:**
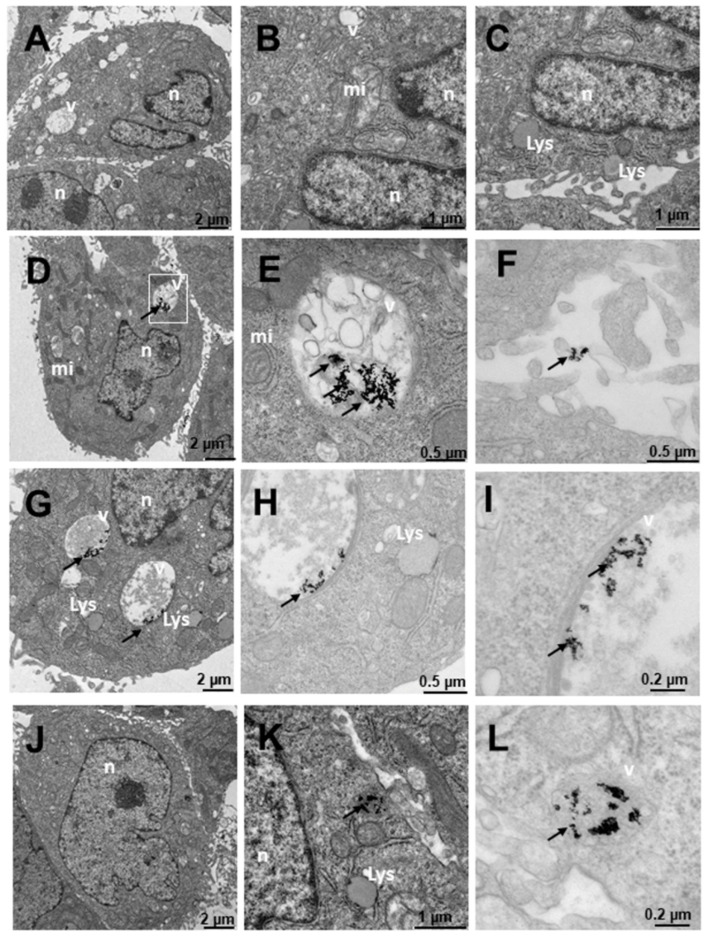
Transmission electron microscopy (TEM) photomicrographs of control cells without treatment (**A**–**C**), compared to Au@16-Ph-16 (N_3_) (**D**–**F**), Au@16-Ph-16/DNA/Doxo (C_3_) (**G**–**I**) and Au@16-Ph-16/DNA/Doxo + Au@16-Ph-16 (C_3_ + N_3_) (**J**–**L**) at 24 h, in HepG2 cells, derived from non-tumorigenic hepatoblastoma, considered as control cells. The dense bodies indicated by an arrow are compatible with the gold core of the nanoparticles. Abbreviations: Lys—lysosome, mi—mitochondria, n—nucleus and v—vesicles.

**Figure 13:**
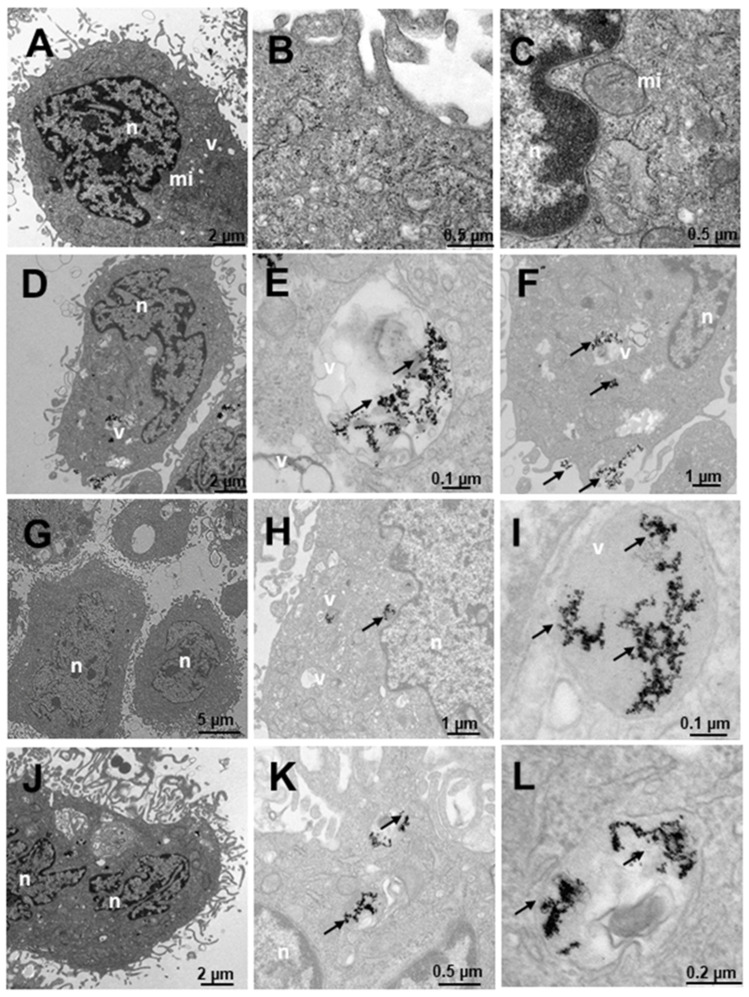
Transmission electron microscopy (TEM) photomicrographs of control cells without treatment (**A**–**C**), compared to Au@16-Ph-16 (N_3_) (**D**–**F**), Au@16-Ph-16/DNA/Doxo (C_3_) (**G**–**I**) and Au@16-Ph-16/DNA/Doxo + Au@16-Ph-16 (C_3_ + N_3_) (**J**–**L**) at 24 h, in SNU387 cells, derived from liver tumor, a model of high-grade hepatocarcinoma. The dense bodies indicated by an arrow are compatible with the gold core of the nanoparticles. Abbreviations: mi—mitochondria, n—nucleus and v—vesicles.

To study the internalization of the different treatments, a transmission electron microscopy study was carried out. For this study, cells were fixed after a 24 h treatment, to ensure that the nanosystems had entered the cells [38]. The study was carried out with Au@16-Ph-16 (N_3_), Au@16-Ph-16/DNA/Doxo (C_3_) and Au@16-Ph-16/DNA/Doxo + Au@16-Ph-16 (C_3_ + N_3_) at 24 h, compared to the control without treatments in non-tumorigenic prostate PNT2 cells (Figure 9), considering control cells, prostate tumor-derived LNCaP cells sensitive to hormone therapy (Figure 10) and DU145 tumor cells derived from prostate tumor, a model of prostate cancer resistant to castration and not responsive to hormonal therapy (Figure 11). Additionally, the internalization in HepG2 cells, derived from non-tumorigenic hepatoblastoma, considering control cells (Figure 12) and SNU387 cells (Figure 13), derived from liver tumor, and a model of high-grade hepatocarcinoma, is studied. This microscopy technique allows the visualization of the organelles and can be used to verify the presence or absence of nanoparticles inside the cells.

Next, measurements were made of the intracellular elements compatible with the gold nuclei belonging to the internalized nanosystems (Table 3). No significant differences between sizes within cells were observed, but there were significant differences with the nanoparticles measured after synthesis, whose gold core had an average size of 3.71 ± 1.18 nm.

To implement the results, quantification of nanoparticles inside cells was performed with ImageJ. The results obtained are shown in Table 4.

The number of cores compatible with the gold nucleus of the precursors and nanosystems (N_3_, C_3_ and C_3_ +N_3_, respectively) were counted. To this end, TEM studies were carried out with ImageJ software using a total of 40,015 nanoparticles from among the five types studied. The results show that the cancer cells internalized a greater number of nanosystems compared to their respective controls in all treatments (see Table 4). Note that for each system, we explored a fixed concentration of N_3_, C_3_ and C_3_ + N_3_ nanosystems; therefore, the number of nanoparticles internalized in each cell line can be compared directly. Thus, for instance, control cells for prostate cancer (PNT2) showed a value for N_3_ of 415 ± 50, compared to cancer cells (LNCaP and DU145) with values of 947 ± 49 and 968 ± 67, respectively. Likewise, for C_3_, the values for PNT2 control cells were 87 ± 25 versus those corresponding to cancer cells (LNCaP and DU145), which were 605 ± 15 and 212 ± 8, respectively. In the case of treatment with C_3_ +N_3_, the values were 214 ± 8 for the control and 790 ± 30 and 802 ± 15 for carcinogens, showing good selectivity in accordance with viability results.

For the study of liver cancer, internalized nanoparticles are higher in cancer cells (SNU387) than control cells (HepG2) in all cases, although in the case of C_3_ + N_3_ the values are only slightly higher in cancer cells. Therefore, these results show how cells with high metabolic activity in general internalize more of both the precursors and the nanosystems studied, in agreement with previous viability results.

Furthermore, to complete the internalization studies, an assortment of confocal microscopy images was collected, showing the presence of NPs within the cells, confirming the TEM results (Figures S16 and S17).

Figure 9 shows the results of PNT2 cells derived from non-tumor prostate, a preneoplastic, non-tumorigenic prostate model. In untreated cells, the appearance is normal with vesicles, mitochondria, nuclei and other organelles also having a normal appearance with villi around the cells (Figure 9A–C). The cells treated with N_3_ (Figure 9D–F) show nanoparticles that are aggregated and internalized in vesicles (Figure 9D–F). Furthermore, nanoparticles can be observed around the cell villi (Figure 9D). To ensure the presence of dense particles compatible with gold nanoparticles and to effectively distinguish them from other particles, low contrast photos were taken in which small elements, such as ribosomes, were blurred but the dense particles remained clearly visible. The vesicles are compartmentalized (Figure 9E) and the number of nanoparticles is high, as can be seen in Figure 9F in greater detail. These vesicles are disposed away from the nucleus (Figure 9D). Cells treated with C_3_ (Figure 9G–I) have nanoparticles arranged in multivesicular bodies, especially near the nucleus. The observed vesicles containing nanoparticles are of two types: endosome-type vesicles and lysosome-type vesicles. The latter were possibly early lysosome types, so it could be deduced that they would later become late endosomes through one of the vesicular communication pathways in animal cells [89]. This would be compatible with the possible elimination of gold debris from the nanoparticles at what is considered the end of the endocytic pathway. Nanoparticles are not observed on the periphery adhering to the villi. The cells treated with C_3_ + N_3_ (Figure 9J–L) present nanoparticles internalized in vesicles (Figure 9J,K) and adhered to the villi (Figure 9J,L). The appearance of the cells is similar to the untreated control cell, differing in number and size of some vesicles. Nanoparticles are found aggregated within the vesicular bodies both near the nucleus and further away from it (Figure 9J).

Figure 10 shows the results of the internalization of LNCaP cells derived from prostate tumor, a hormone-sensitive prostate cancer (HSPC: Hormonse-Sensitive Prostate Cancer) model, which responds to the androgens and secretes prostate specific antigen (PSA). In general, numerous vesicles are observed in the untreated cells in Figure 10A–C, some being large and others very large. Numerous mitochondria can also be observed, which would be related to their high metabolism, and the nucleus appears normal. The cells treated with N_3_ (Figure 10D–F) present nanoparticles adhered to the villi (Figure 10D,F) and internalized in endosome-type vesicles (Figure 10E), those outside the cell being more abundant (Figure 10D,F). The cells treated with C_3_ (Figure 10G–I) internalize the nanoparticles in vesicles compatible with multivesicular bodies and in lysosomes (Figure 10G–I). Nanoparticles are found adhered to the villi, apparently in a smaller quantity than those treated with N_3_ (Figure 10D,F). In cells treated with C_3_ + N_3_ (Figure 10J–L), we observed many vesicles with much cellular debris and nanoparticles, both adhered to villi (Figure 10J–L) and internalized in vesicles (Figure 10J,K). The vesicles are multivesicular and some are very large (Figure 10J,K).

Figure 11 shows the results of the internalization in DU145 cells derived from prostate tumor, a model of castration-resistant tumorigenic prostate cancer (does not respond to hormonal therapy; more aggressive than LNCaP). Both in the cells without treatment (Figure 11A–C) and in those that were treated (Figure 11D–L), cells with many mitochondria are observed, compatible with their high activity, in addition to having numerous villi around them. Several treatments were carried out to observe the internalization of the nanosystem; it was observed that the cells treated with N_3_ (Figure 11D–F) showed dense bodies around the villi attached to them (Figure 11D) and very close to the membrane. Inside the cell, the dense bodies of the gold nanoparticles are arranged in vesicles (Figure 11E–F), especially multivesicular endosomal vesicles, which is the result of endocytosis or incorporation of molecules encompassed by membranes in the endocytosis route, where vesicles formed in the plasma membrane fuse with endosomes. In this case, the said endosomes are further away from the nucleus. In cells treated with C_3_ (Figure 11G–I), the cells present many multicompartmentalized vesicles compatible with endosomes in transition pathways between early endosomes and late endosomes, where dense bodies are located, compatible with the gold nuclei of the nanoparticles. These large nanoparticle vesicles are arranged closer to the nucleus than those treated with N_3_ (Figure 11G). In the experiments carried out with the incorporation of N_3_, after 6 h of treatment with C_3_ (Figure 11J–L), the nanoparticles were arranged both around the nucleus and closer to the plasma membrane (Figure 11K,L), as well as on the periphery of the cells attached to the external villi (Figure 11J). The vesicles that contain the nanoparticles are of two types, we have endosomes and lysosomes (Figure 11J,L). Therefore, we could deduce that after moving to late endosomes, these nanoparticle-loaded vesicles fuse with the lysosomes on one of the vesicular communication pathways that are established in animal cells; this would be compatible with the possible elimination of gold remains at what is considered the end of the endocytic pathway.

Figure 12 shows the internalization of HepG2-like cells derived from non-tumorigenic hepatoblastoma, liver cells. In untreated cells (Figure 12A–C), numerous mitochondria with more abundant large and small vesicles are observed in the area furthest from the nucleus; the rest of the organelles appear normal. Cells treated with N_3_ (Figure 12D–F) present nanoparticles included in multivesicular bodies arranged further away from the nucleus (Figure 12D–E) and very abundantly on the periphery adhered to the villi (Figure 12D,F). Cells treated with C_3_ (Figure 12G–I) present only nanoparticles internalized in the cells in large multivesicular bodies near the nucleus (Figure 12D–F). In the experiments carried out with the incorporation of N_3_, after 6 h of treatment with C_3_ (Figure 12J–L), the nanoparticles were arranged both near the nucleus and in the cytoplasm included in vesicles (Figure 12J–L).

Finally, Figure 13 shows SNU387 cells derived from liver tumor, a high-grade (G4) tumorigenic hepatocarcinoma model. Untreated cells (Figure 13A–C), present many small vesicles (Figure 13A) and many mitochondria, and the appearance of the rest of the organelles is normal (Figure 13A). The cells treated with N_3_ present many vesicles with nanoparticles (Figure 13D–F), arranged towards the periphery compatible with mature phagosomes (Figure 13D–F). The nanoparticles are aggregated within the vesicles and are also attached to the villi around the cells (Figure 13D,F), the latter being fewer in number. C_3_-treated vesicles (Figure 13G–I) present lysosome-compatible vesicles (Figure 13G–H) containing nanoparticles and multivesicular vesicles near the nucleus (Figure 13G–I). Nanoparticles adhered to the villi are not observed (Figure 13G–I). Cells treated with C_3_ + N_3_ (Figure 13J–L) present nanoparticles in vesicles, arranged both around the nucleus and closer to the plasma membrane (Figure 13J,K), as well as on the periphery of cells adhered to the external villi (Figure 13J–K), in fewer numbers than those treated only with N_3_.

**Figure 14:**
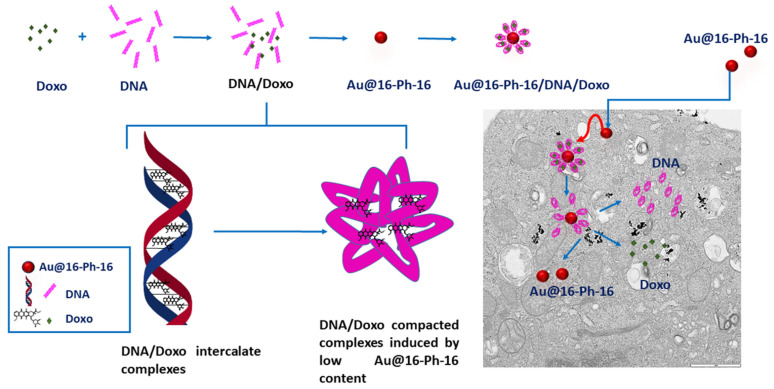
Scheme showing the synthesis of gold nanosystems with Doxo, DNA and gemini surfactants (Au@16-Ph-16/DNA–Doxo), and how the compaction/decompaction processes occur in DNA–Doxo complexes induced by Au@16-Ph-16. The formation of intercalated DNA/Doxo complexes occurs first, followed by the formation of tight DNA/Doxo complexes induced by low Au@16-Ph-16 content. Finally, the internalization of compacted nanocomplexes occurs within the cell and the intracellular release of Doxo by administration of Au@16-Ph-16.

Therefore, the results obtained with TEM studies on C_I_ and N_I_ samples showed that small size spherical nanoparticles usually enter and exit the cell more efficiently [16,90]. Thus, the high degree of internalization obtained for the nanosystems used was in line with previous research on the cellular uptake of different nanoparticles [38]. The effects of these nanosystems on the cells are reflected in the data presented on cell viability, where they are enhanced for the smaller C_1_ and C_1_ + N_1_ (Figure 14). Moreover, nanoparticles loaded to DNA can be seen in Figure S18 demonstrating that Au@16-pH-16/DNA–Doxo nanocomplexes can also be internalized by different type of cells.

Accurate tumor targeting is an important problem to overcome for effective cancer treatment. In fact, drug delivery to the tumor site is crucial to avoid side effects during cancer therapy. In this sense, the use of DNA biomolecules in nanostructures decoration confers great advantages in targeting organelles and biodistribution, promoting the early diagnosis and precise therapy of human cancers [91,92]. Given that DNA is a genetic material possessing high biocompatibility and low cytotoxicity, it is ideal for applications in biomedicine [93,94,95]. Moreover, DNA nanostructures could easily be internalized within the cells and used effectively in a compact form for drug delivery purposes [96]. More specifically, different DNA nanostructures have been demonstrated to be highly effective in delivering Doxo in previous studies carried out under in vivo and in vitro conditions [97,98]. The high loading efficiency of Doxo with different DNA nanostructures relies on the intercalation properties of Doxo within DNA base pairs [97,98,99], with more than 70% of loading efficiency in the specific case of DNA tube structures [97]. Besides loading efficiency, another important issue to be controlled for the effective delivery of Doxo is the controlled release of the drug from the nanostructure. In this sense, the release parameters are clearly dependent on the morphology of the DNA nanostructures [93]. For instance, in the case of tetrahedral, icosahedral and tube DNA forms, the release of Doxo occurs in approximately 10 h, whereas it takes more than 48 h for triangular DNA forms. In this study, the strategy for Doxo delivery is based on the obtaining of compact DNA strands within a DNA nanostructure, contributing to greater stability against DNA–degrading enzymes [100]. This configuration combined with the use of the surfactant precursor, Au@16-Ph-16, as a decompacting agent could modulate biodistribution, clearance time and Doxo release more effectively. Another advantage of the configured nanosystem is related to its biocompatibility, that is, the nontoxicity and non-accumulation in principal organs of Au@16-Ph-16, which integrates the Au@16-Ph-16/DNA–Doxo nanosystem, which was assessed in a previous work; this is crucial to guarantee its possible medical use [40].

Another important aspect is the potential that these nanosystems offer for possible medical applications due to their high positive or negative charge, since they are more stable and, therefore, more effective in their possible applications. Moreover, with reference to the data obtained, these small nanoparticles enter and leave the cell more efficiently, ensuring an adequate effect of the nanosystems on their target cells and facilitating a reduction in the toxicity of systemic treatments with free anticancer agents. Therefore, the general side effects in patients may be reduced, which could influence the achievement of a more favorable general condition after treatment and very possibly a faster recovery.

In summary, TEM images highlight the correct uptake of the distinct nanoformulations studied here, where small size spherical nanoparticles usually enter and exit the cell more efficiently, in accordance with previously published results. However, differences in the degree of uptake are shown by quantifying the number of nanoparticles inside the cells, with the uptake being more evident in cancer cells for N_3_, C_3_ and the combined nanosystems N_3_+C_3_. More specifically, in LNCaP and DU145 lines, the two prostate cancer-derived cell lines, as shown in data from Table 4, demonstrate their specificity for this type of cancer line. However, it should be noted that in both the TEM and viability studies, the effect of the addition of free N_I_ is to induce the decompaction of C_I_ complexes, which contain the anticancer drug. This effect is of extreme importance in some cases, since it induces a greater effect within the cells with respect to free Doxo, in such a way that it enhances the effect of the anticancer agent in the short term, and in other cases, depending on the cell line, a protective effect is produced with respect to the toxicity of free Doxo. Therefore, although the effect may be similar to that of free Doxo in some cases, these treatments with nanosystems entail a more direct and localized effect on the target cells, exerting a similar effect when the nanosystem degrades, so that direct action of the anticancer agent is possible. Finally, the concentrations of free Doxo, Ns, Cs and Cs+Ns are the same in each system tested and since compacted systems are generally less toxic than free Doxo at the same concentration, the objective of minimizing side effects derived from systemic treatments due to the administration of anticancer agents would, therefore, be fulfilled, together with the fact that they act directly on their target cells.

## 3. Materials and Methods

### 3.1. Materials

All chemicals used in this work were analytic-grade reagents and used without further purification. Deoxyribonucleic acid sodium salt from calf thymus (DNA), hydrogen tetrachloroaurate (III) trihydrate, sodium cacodylate, and 3-aminopropyltriethoxilane (APTES) were purchased from Sigma-Aldrich-Merck KGaA (Darmstadt, Germany). Doxo was purchased from Merck-Sigma Aldrich (Saint Louis, MO, USA). Sodium tetrahydridoborate (NaBH_4_) was purchased from Panreac Química S.L.U. (Barcelona, Spain). DNA was used without further purification since preliminary experiments showed that purification did not produce changes in experimental results, the average of number of base pairs (bp) was above 12,000 bp. The absorbance ratio at 260 nm and 280 nm of the double stranded DNA stock solutions was found to be between 1.8 and 1.9 (A_260_/A_280_ = 1.87), which indicates no protein contamination [101]. DNA concentrations (C_DNA_) were measured in base pairs by UV–visible spectroscopy at 260 nm from 13,200 M^−1^·cm^−1^ DNA molar absorptivity [102]. All the solutions were prepared with de-ionized and autoclaved water (conductivity being less than 10^−6^ S·m^−1^) at a fixed ionic strength (I) of 1.63 mM. The total concentrations of the Doxo drug, 16-Ph-16 surfactant, gold nanoparticles covered with 16-Ph-16 surfactant (Au@16-Ph-16), and the compacted nanosystem (Au@16-Ph-16/DNA–Doxo) in a working solution are now referred to as C_Doxo_, C_16-Ph-16_, C_Au@16-Ph-16_, and C_Au@16-Ph-16/DNA–Doxo_, respectively.

#### 3.1.1. Cell Lines and Culture Conditions

Two prostate cancer-derived cell lines (LNCaP and DU145) and one hepatocellular carcinoma-derived cell line (SNU-387), as well as a non-tumor prostate (PNT2) cell line and hepatoblastoma cell line (Hep-G2) were used in this study. All cells were obtained from the American Type Culture Collection (ATCC; Manassas, VA, USA), except the PNT2 cells, which were generously supplied by Dr. J. De Bono (London, UK). Cells were cultured according to manufacturer instructions as previously described [82,103]. Briefly, LNCaP, DU-145, PNT2, and SNU-387 cells were cultured on RPMI-1640 (Lonza, Madrid, Spain) supplemented with 10% fetal bovine serum (FBS; Merck KGaA, Damstadt, Germany), 1% L-glutamine (Thermo Fisher Scientific, Madrid, Spain), and 0.2% gentamicin-amphotericin B (Thermo Fisher Scientific), whereas Hep-G2 cells were cultured on MEM (Thermo Fisher Scientific) supplemented with 10% FBS, 1% pyruvate (Thermo Fisher Scientific), and 0.2% gentamicin-amphotericin B at 37 °C in a humidified 5% CO_2_ atmosphere. Cell lines were validated by analysis of short tandem repeats (STRs) sequences using GenePrint 10 System (Promega, Barcelona, Spain) and checked for mycoplasma contamination by PCR as previously reported [104].

#### 3.1.2. Synthesis of N,N’-[1,3-phenylenebis(methylene))bis[N,N’-dimethyl-N-(1-hexadecyl)]-ammonium dibromide, 16-Ph-16

The synthesis of 16-Ph-16 gemini surfactant (see Figure S1) was carried out using α,α’-dichloro-p-xylene (11.9 g, 0.068 mmol) and N,N-dimethylhexadecylamine (40.1 g, 0.15 mmol) as reagents. For this purpose, the xylene derivative was dissolved in dry acetonitrile and added dropwise to a stirred solution of the amine in 150 mL of acetonitrile. The mixture was then refluxed for 96 h, whereupon the solvent was removed under reduced pressure. As a result, a white solid was obtained and recrystallized from a (4/1) acetone/hexane mixture [105,106]. Finally, once the sample was cooled, a white solid was recovered by filtration. All products were recrystallized from ethyl acetate up to five times and dried under vacuum [106]. The critical micelle concentration (CMC) of the synthesized surfactant was measured via the surface tension technique, giving (8.0 ± 0.4) × 10^−6^ M. Upon cooling, a white solid was recovered by filtration.

#### 3.1.3. Synthesis of Au@16-Ph-16 Gold Nanoparticles

To prepare 16-Ph-16 functionalized gold nanoparticles, 300 μL of aqueous solution of 23 mM HAuCl_4_ at 99.9% purity was added to 30 mL of 16-Ph-16 surfactant 4 × 10^−5^ M and the mixture was stirred vigorously for 5 min in the absence of light, giving a yellow solution. Subsequently, 100 μL of a freshly prepared 0.4 M NaBH_4_, 96% pure, aqueous solution was added dropwise to the previously prepared mixture and stirred moderately for 15 min in the darkness, acquiring a reddish color [40]. As a result, an aqueous solution of Au@16-Ph-16 nanoparticles at 5.6 × 10^−8^ M concentration was obtained. In this work, we employed three formulations (N_I_) of Au@16-Ph-16 prepared at different C_16-Ph-16_ concentrations of 3.4, 6.7 and 9.4 nM, which were designated as N_1_, N_2_ and N_3_, respectively. The concentrations of Ns, Cs and Cs + Ns were the same in each system tested.

#### 3.1.4. Synthesis of Au@16-Ph-16/DNA–Doxo Nanocomplexes

The Doxorubicin hydrochloride (Doxo) (Sigma-Aldrich-Merck KGaA, Darmstadt, Germany) transporter was prepared from DNA/Doxo complexes and the appropriate quantity of the synthesized precursor, Au@16-Ph-16, to guarantee the maximum compaction state of the biopolymer in each case. Note that the optimal relative nanoparticle-DNA concentrations were established at R = C_Au@16-Ph-16_/C_DNA_ = 7.5·10^−5^ (see Section 3.1 for more details). Moreover, the DNA/Doxo complexes were prepared under saturation conditions in order to transport the maximum amount of drug per nanocomplex (X = C_Doxo_/C_DNA_ = 0.025) and to ensure the formation of the intercalative DNA/Doxo complex. In this sense, Pérez-Arnaiz et. al. have shown that, depending on the C_Doxo_/C_DNA_ concentrations ratio, two types of complexes can be obtained: (i) an intercalated complex for C_Doxo_/C_DNA_ < 0.3 and (ii) an external complex for C_Doxo_/C_DNA_ > 0.3 [107]. Three compacted formulations (C_I_), designated as C_1_, C_2_ and C_3_, were explored at different C_Doxo_, C_DNA_ and C_Au@16-Ph-16_ concentrations, working at the already established fixed X and R. The concentration of the reactants was C_Doxo_ = 0.25 µM, C_DNA_ = 10 µM and C_Au@16-Ph-16_ = 0.75 nM for C_1_; C_Doxo_ = 0.50 µM, C_DNA_ = 20 µM and C_Au@16-Ph-16_ = 1.5 nM for C_2_; and C_Doxo_ = 0.70 µM, C_DNA_ = 28 µM and C_Au@16-Ph-16_ = 2.1 nM for C_3_.

To obtain stable Au@16-Ph-16/DNA–Doxo complexes, the appropriate concentration of Doxo was added to an aqueous solution of calf thymus ds-DNA at room temperature; the mixture was then gently stirred for 2 min. Subsequently, the appropriate concentration of Au@16-Ph-16 was added to the DNA–Doxo complex, and the mixture was stirred for 5 min. A change in the surface plasmon resonance (SPR) maximum wavelength (λ_MAX_) from 522 nm to 520 nm accompanied by an increase in the absorbance intensity of the precursor was indicative of the formation of the nanocomplexes (see Figure S2). In all systems, the concentrations of free Doxo, Ns, Cs and Cs + Ns were the same in each system tested.

### 3.2. Methods

#### 3.2.1. Cell Viability

Cell viability was evaluated using the Alamar-Blue assay (Bio-Source International, Camarillo, CA, USA) as previously reported [104]. Briefly, cells were seeded in 96-well plates at a density of 2500–5000 cells/well and serum-starved for 24 h. The DNA, Doxo and Au@16-Ph-16 dose for cell viability assay is given in Section 3.1.4. of the paper. Then, for Doxo, C_I_ and N_I_ compounds, an aliquot of 5 µL of each compound was mixed with 95 µL of the culture medium to obtain the final concentration of the reactants. Thus, the final dose of DNA in C_1_, C_2_ and C_3_ compounds before dilution in the culture medium was 0.5 µM, 1 µM and 1.4 µM, respectively. The corresponding final Au@16-Ph-16 dose was as 3.75 pM, 7.5 pM and 10.5 pM for C_1_, C_2_ and C_3_ compounds, respectively. The final Doxo dose was 0.125 µM, 0.25 µM and 0.35 µM for C_1_, C_2_ and C_3_ nanosystems, respectively. Note that the same Doxo dose as in C_I_ compounds was used for Doxo_1_, Doxo_2_ and Doxo_3_ free drug controls. Besides, the dose after mixing of N_I_ compounds was 0.17 nM, 0.34 nM and 0.47 nM, respectively for N_1_, N_2_ and N_3_ nanoparticles. Fluorescence (560 nm) was evaluated using the FlexStation III system and resurzirin as the fluorescence probe (Molecular Devices, Sunnyvale, CA, USA) after 3 h of incubation with the Alamar-Blue compound at 10%. Moreover, the fluorescence of the different N_I_, C_I_, N_I_ + C_I_ and Doxo_I_ compounds of each treatment was evaluated in the absence of cells at the same experimental condition (λ_emission_ = 560 nm) to take into account the fluorescence contribution of each individual compound. The fluorescence contribution of these compunds is given by the Doxo concentration in each nanoformulation and the phenyl ring of the cationic surfactant in N_I_ compounds [38,39,108]. Thus, corrected cell viability was then evaluated at 48 h considering the fluorescence of the medium itself and of each specific system in response to different experimental treatment conditions (for more details, see Section 2 in the Supporting Information).

All in vitro experiments were performed at least 3 different times (n ≥ 3), and with at least 2 technical replicates. Standard error deviation was calculated and represented on the same graph.

#### 3.2.2. UV–Vis Spectroscopy

Absorbance spectra were carried out using a CARY 500 SCAN UV−vis−NIR (ultraviolet/visible/near-infrared) spectrophotometer (Varian, Markham, ON, Canada). Data were collected every 2 nm with a standard quartz cell having a 10 mm path length. The wavelength accuracy and spectral bandwidth were ±0.3 nm and 0.5 nm, respectively. To study the formation of the nanocomplexes, the spectra of the Au@16-Ph-16 nanoparticles at the concentrations used for preparing the compacted nanocomplex, Au@16-Ph-16/DNA–Doxo, were recorded in the wavelength range of 800–400 nm, after 48 h of equilibration (see Figure S2). Moreover, the stability of the N_I_ and C_I_ formulations was checked for 1 month, following the possible changes in UV–Vis spectra from 200 to 800 nm over time (see Figure S3).

#### 3.2.3. Fluorescence Spectroscopy

Fluorescence measurements were carried out at 298.0 K in a Hitachi F-2500 spectrofluorometer (Hitachi High Technologies America, Inc., Pleasanton, CA, USA) interfaced to a PC for reading and handling the spectra. The interaction of Au@16-Ph-16 nanoparticles with the already formed DNA/Doxo complex was studied using fixed C_Doxo_ = 2.5 μM and C_DNA_ = 100 μM concentrations and varying the C_Au@16-Ph-16_ from 0.53 to 128 nM. The excitation and the emission wavelengths were 480 and 563 nm, respectively.

For the release assay, we used the appropriate Au@16-pH-16 concentration to induce the decompaction of the Au@16-Ph-16/DNA–Doxo nanocomplex and release of Doxo. The C_3_ complex was freshly prepared and stabilized for 48 h before the addition of N_3_. After that, the fluorescence of the C_3_ complex increased at first and then stabilized in 60 min, indicating the correct release of Doxo from the complex. The quantity of Doxo released from the C_3_ nanocomplex (drug release: DR) was measured from the stabilized fluorescence spectra of the C_3_ + N_3_ using the appropriate fluorescence calibration curve for free Doxo in the presence of gold nanoparticles (see Supplementary Materials Figure S12). DR was calculated as follows: %DR = (C_Doxo_ released upon addition of N_3_ to C_3_ complex/C_Doxo_ total concentration added for C_3_ preparation) × 100 [74].

Encapsulation efficiency was calculated after the incubation of the prepared C_3_ nanocomplex for 48 h and subsequent dialysis using a cellulose ester dialysis membrane for 24 h (Sigma-Aldrich-Merck KGaA, Darmstadt, Germany, MWCO = 10,000). The fluorescence spectra of the dialyzed samples were recorded in the wavelength range of 500–700 nm. The amount of Doxo loaded on the nanoparticles was assessed by measuring the fluorescence of C_3_ at 560 nm and after correction from Au@16-Ph-16 contribution (see Supplementary Materials Figure S13). Herein, the encapsulation efficiency (EE) was calculated as follows: %EE = (C_Doxo_, _total added_ − C_Doxo_, _free in_ C_3 nanocomplex_)/C_Doxo_, _total added_) × 100 = (0.70 µM − 0.058 µM)/0.70 µM = 92% [74].

#### 3.2.4. Circular Dichroism (CD) Spectroscopy

Electronic CD spectra were recorded with a BioLogic Mos-450 spectropolarimeter (Barcelona, Spain). A standard quartz cell with a 10 mm path length was used. The spectra were expressed in terms of molar ellipticity, [θ]. Scans were taken from 220 nm to 320 nm, working in the intrinsic CD region of DNA. For each spectrum, 5–10 runs were averaged at a constant temperature of 298.0 K with a 10 min equilibration before each scan. The interactions and conformational changes induced by the Au@16-Ph-16 nanoparticles in DNA/Doxo complexes were studied working at fixed C_DNA_ = 200 μM and C_Doxo_ = 5 μM concentrations and varying Au@16-Ph-16 concentrations, from 0.0053 nM to 21.3 nM.

#### 3.2.5. Atomic Force Microscopy Experiments

The AFM images were obtained with a Molecular Imaging Picoscan 2500 microscope (Agilent Technologies, Las Rozas, Madrid, Spain). For imaging in air, silicon cantilevers (Model Pointprobe, Nanoworld Neuchâtel, Switzerland) with a resonance frequency of approximately 240 kHz and with a spring constant of 42 N/m were used. All AFM images were recorded in tapping mode, with scan speeds of about 0.5 Hz and data collection at 256 × 256 pixels. The acquired AFM images were flattened to remove the background slope [109]. To ensure the correct sample visualization, the mica surface was first modified and incubated for 20 min with a 0.1% (*v*/*v*) APTES solution. Subsequently, the surface was washed with ultrapure water and air dried. A total of 30 μL of isolated DNA (C_DNA_ = 0.3 μM), the Doxo/DNA complex (C_DNA_ = 0.3 and C_Doxo_ = 7.5 nM) or the Au@16-Ph-16/DNA–Doxo nanocomplexes (C_DNA_ = 0.3 μM and C_doxo_ = 7.5 μM) at different C_Au@16-Ph-16_ and R ratios (R = 3.6 × 10^−6^ − 4.2 × 10^−4^) was dropped onto this modified surface. Note that for imaging DNA and different DNA complexes, the C_DNA_ was adjusted and diluted due to the large size of these biopolymers, ensuring that the molecules were spread over the surface with no overlap. The adsorption time of the prepared samples was 30 min. The sample was then thoroughly rinsed with doubly distilled water and finally air dried for AFM imaging.

#### 3.2.6. Dynamic Light Scattering (DLS) and Zeta Potential Measurements

The size and distribution of the synthesized N_I_ and C_I_ nanoformulations described in Section 3.1.3 and Section 3.1.4 were characterized by means of the DLS technique using a Zetasizer Model ZS-90 (Malvern, Worcestershire, UK). The samples were illuminated with a laser characterized by a fixed detection arrangement of 90° to the center of the cell area, and the fluctuation in intensity of the scattered light was then analyzed. At least 5 size measurements were taken for each sample and the relative error for the hydrodynamic diameter was calculated to be <5%. Zeta-potential (ζ) values were obtained measuring the electrophoretic mobility of the sample from the velocity of the particles using a laser Doppler velocimeter (LDV). A Zetasizer Nano ZS from Malvern Instrument Ltd. (Worcestershire, UK) was used. At least six zeta-potential measurements were taken for each sample by using a DTS1060 polycarbonate capillary cell.

#### 3.2.7. Transmission Electron Microscopy (TEM)

To examine the size and morphology of the isolated Au@16-Ph-16 gold nanoparticles, a copper grid coated with a carbon film was used. The grid was allowed to air dry for several hours at room temperature. TEM analysis was performed with a high-resolution TEM-TALOS F200S electron microscope. The resulting images were analyzed using ImageJ 1.52a software. From these measurements, Au@16-Ph-16 was found to have a diameter of (3.7 ± 1.2) nm (see Figure S4). With the same software, a count of the nanoparticles inside the cells was carried out, for a total of 40,015 nanoparticles from among the five types used. For visualization of the precursor and compacted nanosystems in cell samples, a Zeiss electron microscope was used. Approximately 500 cells were visualized, with anticancer treatment with Doxo, free Au@16-pH-16 (N_3_) nanoparticles and Au@16-Ph-16/DNA–Doxo compact nanosystems (C_3_), as well as their nanosystem controls alone. The different cell groups were fixed in 1.6% glutaraldehyde. They were then washed in cacodylate trihydrate solution (0.1 M and pH: 7.4) for 1 h at room temperature and/or 277.0 K overnight, then placed in the Automatic Sample Processor with a 33 h and 25 min protocol. The samples were then post-fixed with a 1% osmium tetroxide solution and counterstained with a 2% uranyl acetate solution to contrast the sample. Subsequently, the samples were dehydrated and gradually embedded in epoxy resin. Finally, they remained at 343.0 K for 7 h for polymerization of the resins. We then proceeded to first perform semi-fine cuts, with a glass sheet in a standard range of 300 nm. To determine the study areas, prior to making the ultra-fine sections, semi-fine sections were made and stained with toluidine blue, and then visualized with an optical microscope. Ultra-fine cuts were then made with a diamond disc, less than or equal to 70 nm. The visualization of samples was carried out with a Zeiss Libra microscope, using 300 copper mesh grids. For more details, see the protocol followed by the research group in previous studies [38,40].

#### 3.2.8. Energy Dispersive Spectroscopy (EDS) Measurements

In order to study of the elemental components of the prepared cells fixed, treated and cut in an ultramicrotome, and to check the presence of the nanoparticle in the sample, a microanalysis was performed on an ultra-fine section in the Zeiss EVO LS15 scanning electron microscope using energy dispersive spectroscopy (EDS) for the chemical analysis of the sample.

#### 3.2.9. Inductively Coupled Plasma Mass Spectrometry (ICP-MS)

The concentration of Au in the samples was determined by inductively coupled plasma mass spectrometry (ICP-MS) (8800 ICP-MS/MS from Agilent, Agilent Technologies, CA, USA) after pretreatment by microwave digestion (Ethos One, Milestone Rsl, BG, Italy), with doubly distilled nitric acid, obtained in-house and used with a sub-boiling purification system (DST-1000 from Savillex, MN, USA). A semiquantitative analysis was performed. According to the manufacturer, the uncertainty of semiquantitative results is approximately 30%. Due to technical requirements, for the analysis, it was necessary to collect a pellet of approximately 0.5 g from each cell line used in the sampling for analysis.

#### 3.2.10. Confocal Microscopy

Cells were seeded into glass coverslips at a density of 150,000–300,000 cells per glass. After 24 h of treatment, cells were fixed with 4% paraformaldehyde for 30 min at RT. After fixation, cells were incubated with 4′,6-diamidino-2-phenylindole (DAPI 1 µg/mL; D9542) (Sigma-Aldrich-Merck KGaA Darmstadt, Germany) for 1 h at RT. After washing, coverslips were mounted on microscope slides and examined with confocal microscopy using a super-resolution Zeiss LSM 880 upright confocal microscope with Airyscan (Carl Zeiss NTS GmbH, Oberkochen, Germany). The LSM 880 system has 8 laser lines and a range of ultra-sensitive detectors, including the Airyscan detector module, that are run through the Zen 2 LSM software platform. Images were processed using Zen Black and Zen Blue software and a 60X/oil objective.

## 4. Conclusions

The strategy presented attempts to transport anticancer drugs using compacted gold nanocomplexes with DNA as a vehicle. This configuration based on the use of low size nanosystems, whose mean diameters vary between 33 and 57 nm, leads to the achievement of drug nanocarriers with promising projections in healthcare. As has been meticulously described, the results obtained show the viability of the proposed methodology for the internalization of compacted nanocomplexes inside the cell, as well as the effectiveness and selectivity of the C_I_ + N_I_ treatments against cancer cells. In addition, the direct action of the nanocomplexes on the target cancer cells, as well as the protective effect in non-tumor cells, would contribute to minimizing the side effects derived from systemic treatments based on the use of the free drug.

## Figures and Tables

**Table 1:** Zeta potential of Au@16-Ph-16 precursors (N_I_) and Au@16-Ph-16/DNA–Doxo compacted nanosystems (C_I_) at different concentrations in water and PBS 0.1X (I = 1.63 mM, pH = 7.4).

System	Zeta Potential/mV in Water	Zeta Potential/mV in PBS 0.1X
**N_1_**	(30 ± 3)	(18.1 ± 1.7)
**N_2_**	(34 ± 3)	(28 ± 3)
**N_3_**	(37.0 ± 0.9)	(40.4 ± 0.7)
**C_1_**	(−49 ± 6)	(−28 ± 5)
**C_2_**	(−49 ± 3)	(−44 ± 5)
**C_3_**	(−35.5 ± 1.2)	(−30 ± 3)

**Table 2:** Dynamic light scattering (DLS) size distribution by number of Au@16-Ph-16/DNA–Doxo compacted nanosystems (C_I_) at different concentrations in water and PBS 0.1X (I = 1.63 mM, pH = 7.4).

System	Diameter/nm in Water	Diameter/nm in PBS 0.1X
**C_1_**	(33 ± 7)	(67 ± 11)
**C_2_**	(57 ± 11)	(71 ± 11)
**C_3_**	(44 ± 7)	(64 ± 9)

**Table 3:** Diameters of elements internalized in cells compatible with core AuNPs.

Treatment	PNT2	LNCaP	DU145	HepG2	SNU387
Au@16-Ph-16 (N_3_)	5.9 ± 1.1	6.2 ± 1.6	5.3 ± 1.4	5.6 ± 1.0	5.8 ± 1.0
Au@16-Ph-16/DNA/Doxo (C_3_)	5.8 ± 1.4	6.4 ± 1.3	5.78 ± 1.4	5.9 ± 1.0	5.9 ± 0.9
Au@16-Ph-16/DNA/Doxo + Au@16-Ph-16 (C_3_ + N_3_)	6.7 ± 1.1	6.3 ± 1.5	5.9 ± 0.8	6.1 ± 0.9	6.,6 ± 1.1

**Table 4:** Number of internalized nanoparticles per treatment in each type (Mean ± SD).

Treatment	PNT2	LNCaP	DU145	HepG2	SNU387
N_3_	415 ± 50	947 ± 49	968 ± 67	678 ± 52	761 ± 44
C_3_	87 ± 25	605 ± 15	212 ± 8	48 ± 5	63 ± 8
C_3_ + N_3_	214 ± 8	790 ± 30	802 ± 15	662 ± 84	671 ± 27

## Data Availability

Not applicable.

## Supplementary Materials

The following supporting information can be downloaded at: https://www.mdpi.com/article/10.3390/ijms232415575/s1, 1: Result of Characterization of p-16-Ph-16 cationic gemini surfactant; Figure S1: Combined COSY and spectra of compound m-16-Ph-16 together with the respective 1H NMR spectrum on both axes and Structure of the compound p-16-Ph-16. 2. Protocol Cell Viability Correction; Figure S2: Absorbance spectra of the precursor and compacted nanocomplexes evidencing the formation of the complexes in a cacodylate buffer; Figure S3: Stability for different formulations over 0 days at 1 month; Figure S4: TEM image of free Au@16-Ph-16 gold nanoparticles and size distribution of Au@16-Ph-16 in water; Figure S5: Zeta potential of Au@16-Ph-16 nanoparticles at different C_Au@16-Ph-16_ concentrations in water; Figure S6: Zeta potential of Au@16-Ph-16/DNA-Doxo compacted nanocomplexes for different formulations in water; Figure S7: Zeta potential of Au@16-Ph-16 nanoparticles at different C_Au@16-Ph-16_ concentrations in PBS 0.1X; Figure S8: Zeta potential of Au@16-Ph-16/DNA-Doxo compacted nanocomplexes for different formulations in PBS 0.1X; Figure S9: DLS size distribution by number of Au@16-Ph-16/DNA-Doxo compacted nanocomplexes for different formulations in water; Figure S10: DLS size distribution by number of Au@16-Ph-16/DNA-Doxo compacted nanocomplexes for different formulations in PBS 0.1X; Figure S11: Intro release profile of Doxo from C_3_ compacted nanosystems upon addition of N_3_ precursor; Figure S12: Release assay for C_3_ nanocomplex. C_3_ release in the presence of N_3_ nanoparticles and Doxo calibration curve used for measuring released-drug in the presence of N_3_; Figure S13: Loading assay for C_3_ nanocomplex. The spectra of C_3_ system is displayed upon 48 h of stabilization and after correction with Au@16-Ph-16 contribution. C_3_ loading and Doxo calibration curve used for measuring encapsulation efficiency; Figure S14: Corrected Cell Viability of treated cells versus control at 48 h after treatment with Doxo_1_, C_1_, C_1_+N_1_ and N_1_ formulations. HepG2 and SNU387 liver cell lines, PNT2, LNCap and DU145 cell lines; Figure S15: Corrected Cell Viability of treated cells versus control at 48 h after treatment with Doxo_2_, C_2_, C_2_+N_2_ and N_2_ formulations. HepG2, SNU387 liver cell lines, PNT2, LNCap and DU145 cell lines; Figure S16: Confocal microphotographs showing cells treated for 24 h with Au@16-Ph-16 (N_3_) and Au@16-Ph-16/DNA/Doxo + Au@16-Ph-16 (C_3_ + N_3_), in PNT2 and DU145 prostate cells; Figure S17: Confocal microphotographs showing cells treated for 24 h with Au@16-Ph-16 (N_3_) and Au@16-Ph-16/DNA/Doxo + Au@16-Ph-16 (C_3_ + N_3_), in HepG2 and SNU387 liver cells; Figure S18: Photomicrographs (TEM) of different tumorigenic and non-tumorigenic cells treated with Au@16-Ph-16/DNA-Doxo indicating the DNA biopolymer.
