# Supplementary material for: Gold Nanosystems Covered with Doxorubicin/DNA Complexes: A Therapeutic Target for Prostate and Liver Cancer
Source: Int J Mol Sci. 2022 Dec 8;23(24):15575. doi: 10.3390/ijms232415575 (PMC9779246; doi:10.3390/ijms232415575)
Supplement: Supplementary file 1 [file ijms-23-15575-s001.zip › ijms-2014935-supplementary.pdf]

Supporting Information

Gold nanosystems covered with doxorubicin/DNA complexes. Therapeutic target for the prostate and liver cancer

Rosa M. Giráldez-Pérez, [*a#] Elia Grueso, [*b#] Antonio J. Montero-Hidalgo, [c] Raúl M. Luque, [c] José María Carnerero [b], Edyta Kuliszewska [d] and Rafael Prado-Gotor [b]

University of Cordoba, Faculty of Sciences, Department of Cell Biology, Physiology and Immunology, 14014 Cordoba, Spain.[a]

University of Seville, Faculty of Chemistry, Department of Physical Chemistry, 41012 Seville, Spain.[b]

Maimonides Biomedical Research Institute of Cordoba (IMIBIC); Reina Sofia University Hospital (HURS); Department of Cell Biology, Physiology and Immunology, University of Cordoba; CIBER Physiopathology of Obesity and Nutrition (CIBERobn) 14004 Cordoba, Spain[c]

Chemtra, 47-300 Krapkowize, Poland.[d]

[#] Rosa M. Giráldez-Pérez and Elia Grueso contributed equally to this paper.

[*] Authors to whom correspondence should be addressed. E-mail: rgiraldez@uco.es, elia@us.es

1. Characterization of p-16-Ph-16 cationic gemini surfactant

NMR Characterization

***N,N′*-Di-*n*-hexadecyl-*N,N,N′,N′*-tetramethyl-phenylene-1,4-dimethylenammonium dibromide (p-16-Ph-16).** ^{1}H-NMR (400 MHz, $CDCl_3$, δ in ppm): 7.79 (s, br, 4 H, H-2 (4x)); 5.10 (s,br, 4 H, H-1′ (2x)); 3.51 (s,br, 4 H, H-1′′ (2x)); 3.21 (s,br, 12 H, CH_3 (4x)); 1.80 (s,br, 4 H, H-2′′ (2x)); 1.35 (s,br, 4 H, H-3′′ (2x)); 1.24$_{c}$ (m,br, 48 H, H-4′′ to H-15′′ (2x)); 0.68 (t, 6 H, *J*=7.0 Hz, H-16′′ (2x)). ^{13}C NMR (100 MHz, $CDCl_3$, δ in ppm): 133.6 (d, C-2 (4x)); 128.7 (s, C-1 (2x)); 66.6 (t, C-1′ (2x)); 65.1 (t, C-1′′ (2x)); 49.6 (q, CH_3 (4x)); 31.4 (t, C-14′′ (2x)); 29.7-29.4 (t (10x), C-4′′ to C-13′′ (2x)); 28.5 (t, C-3′′ (2x)); 26.4 (t, C-2′′ (2x)); 22.7 (t, C-15′′ (2x)); 14.1 (q, C-16′′ (2x)).

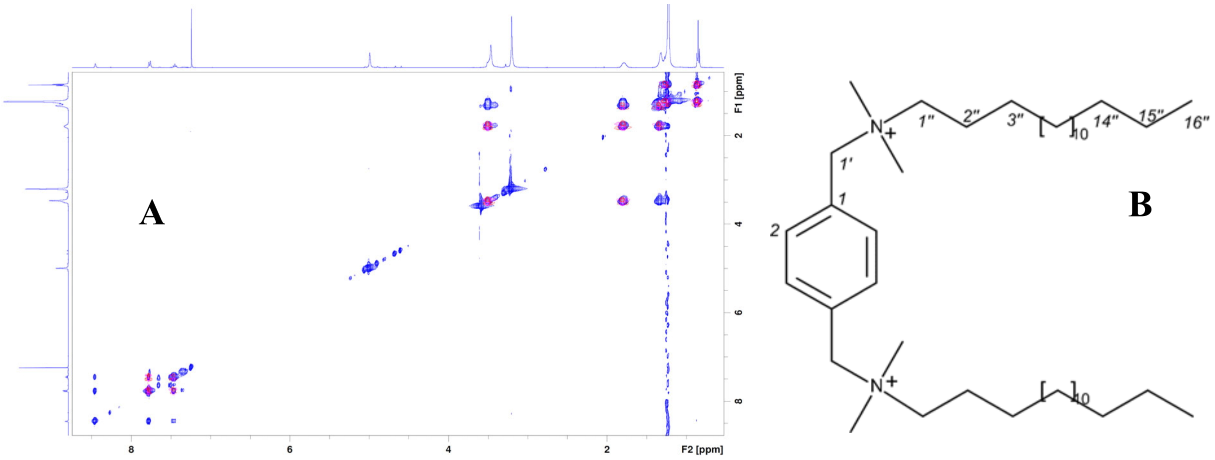

Figure S1. (A) Combined COSY (pink) and TOCSY (blue) spectra of compound m-16-Ph-16 together with the respective ^{1}H NMR spectrum on both axes. **(B)** Structure of the compound p-16-Ph-16. The numbering is in accordance with that used for the NMR shift data. It is not in accordance with IUPAC nomenclature, but allows an easy comparison of different shifts in the compound.

2. Cell Viability Correction

Viability data of different cellular lines were obtained from the registered fluorescence at 560 nm wavelength of each N_I, C_I, N_I+C_I and $Doxo_I$ system in the presence of resazurin at 0 hours and after 48 hours of treatment. The fluorescence results were firstly corrected from the own fluorescence of the culture medium in the absence of cells, that is:

$$I_{0h} = I_{560nm,\ 0h} - I_{560nm,\ medium} \quad (S1)$$

$$I_{48h} = I_{560nm,\ 48h} - I_{560nm,\ medium} \quad (S2)$$

where $I_{560nm,\ medium}$ is the mean fluorescence of the culture medium in the absence of cells; $I_{560nm,\ 0h}$ and $I_{560nm,\ 48h}$ are the mean fluorescence of each compound in the presence of resazurin without the medium correction, and I_{0h} and I_{48h} are the mean fluorescence intensity of the treatment corrected from the contribution of the culture medium at 0 and 48 hours, respectively.

Subsequently, a second correction was needed taking into account the own fluorescence contribution of each N_I, C_I, N_I+C_I and $Doxo_I$ system at 560 nm in the absence of resazurin. As is well-known free doxorubicin exhibit two fluorescence emission maxima at 559 nm and 593 nm in water, which are very close to the fluorescence maximum of resazurin [1]. Moreover, N_1, N_2 and N_3 compounds possess fluorescence spectra with a maximum at 589 nm due to the phenyl ring of the cationic gemini surfactant [2-3]. As a result of these contributions, the viabilities of each compound in different cell lines should be corrected. For instance, if I_{C3} is the fluorescence emission in the absence of cells of C_3 compound at 560 nm, the corrected cell viability for C_3 ($V_{C3,\ corr}$) is calculated as follow:

$$V_{C3,\ corr} = \frac{(I_{48h} - I_{C3})}{(I_{0h} - I_{C3})} \quad (S3)$$

Finally, cell viabilities of treated cells were expressed respect to the viability of the control ($V_{Control}$), for which a 100% of viability was assigned. Hence, the corrected cell viability versus control for C_3 ($V_{C3,\ corr}^{Control}$) can be calculated in accordance with equations 4-5:

$$V_{Control} = \frac{(I_{48h,control})}{(I_{0h,\ control})} \quad \text{(S4)}$$

$$V_{C3,\ corr}^{Control} = \frac{V_{C3,\ corr}}{V_{Control}} \times 100 \quad \text{(S5)}$$

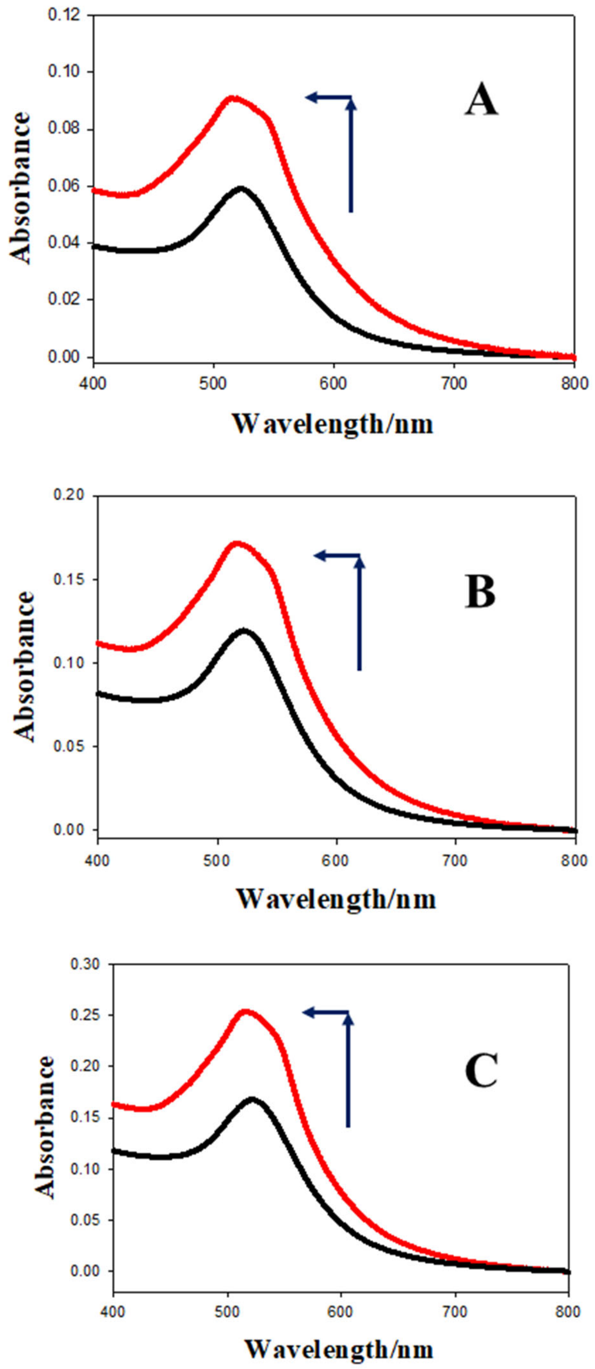

Figure S2. Absorbance spectra of the precursor and compacted nanocomplexes evidencing the formation of the complexes in a cacodylate buffer (I = 1.63 mM and pH = 7.4). (A) N_1 (black) and C_1 (red); (B) N_2 (black) and C_2 (red); (C) N_3 (black) and C_3 (red).

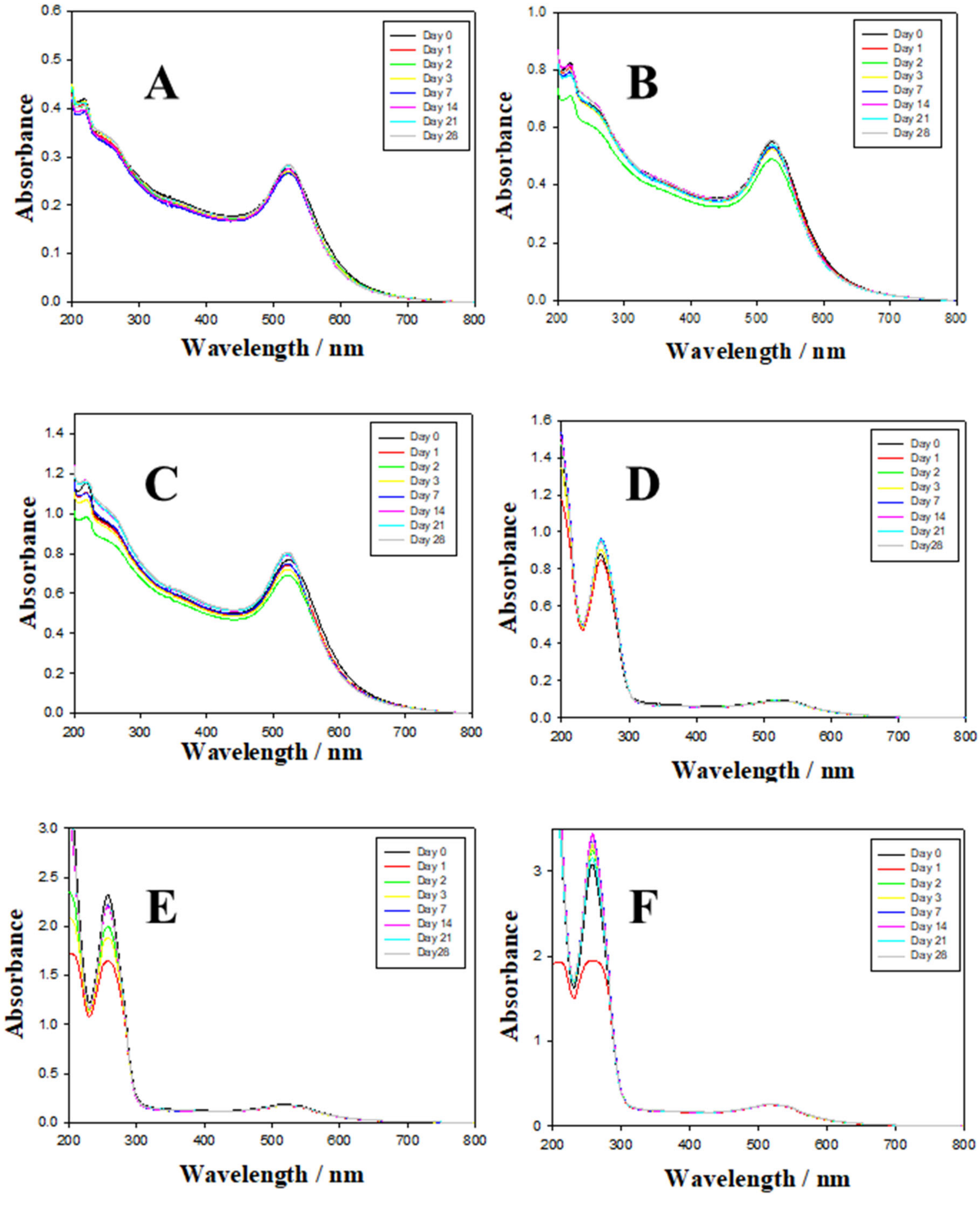

Figure S3. Stability for different formulations over the time: 0 days (black); 24 hours (red); 48 hours (green); 72 days (yellow); 1 week (blue); 2 weeks (pink); 3 weeks (cyan) and 1 month (gray). (A) N_1; (B) N_2; (C) N_3; (D) C_1; (E) C_2 and (F) C_3.

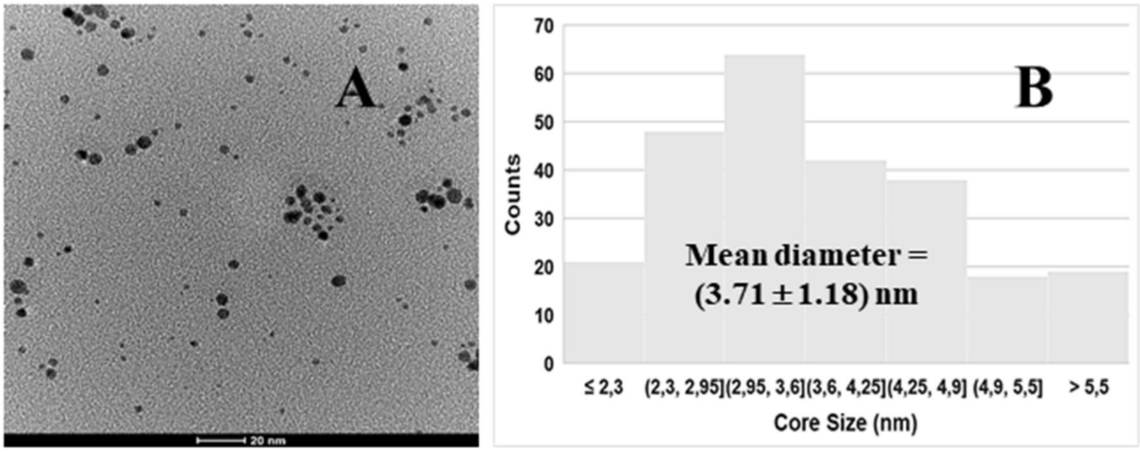

Figure S4. (A) TEM image of free Au@16-Ph-16 gold nanoparticles. (B) Size distribution of Au@16-Ph-16 in water.

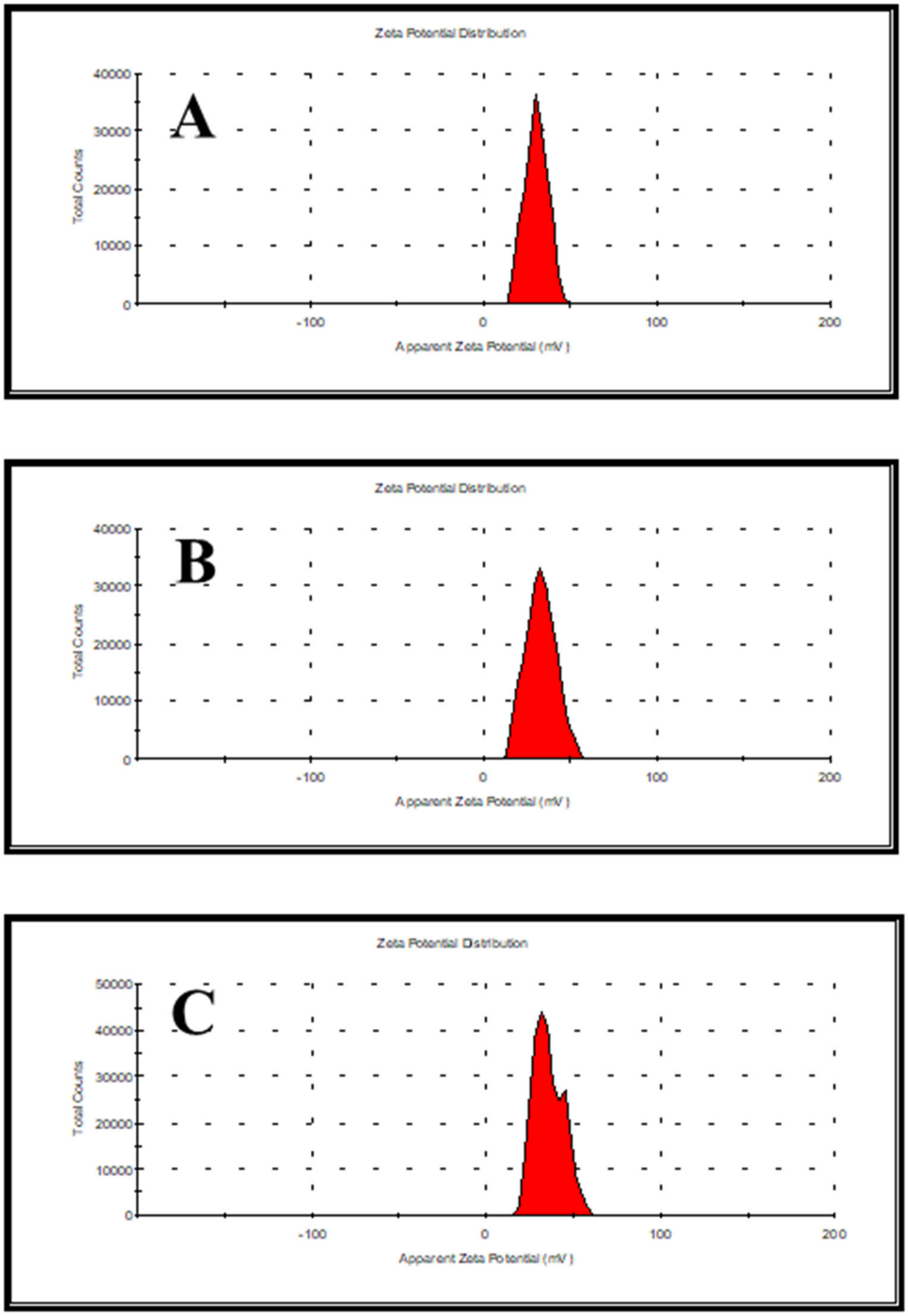

Figure S5. Zeta potential of Au@16-Ph-16 nanoparticles at different $C_{Au@16\text{-}Ph\text{-}16}$ concentrations in water. (A) N_1, (B) N_2 and (C) N_3.

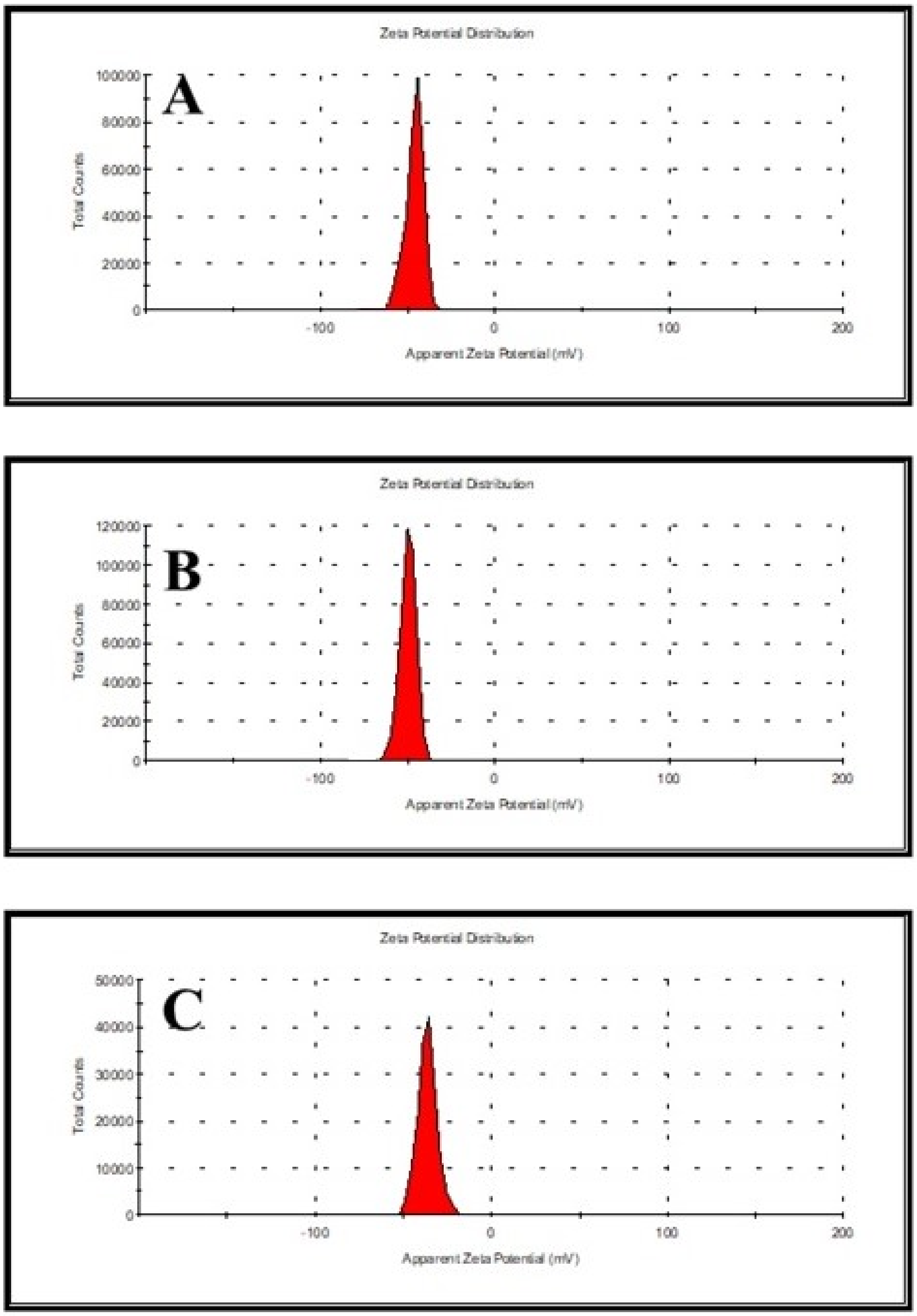

Figure S6. Zeta potential of Au@16-Ph-16/DNA-Doxo compacted nanocomplexes for different formulations in water. (A) C_1, (B) C_2 and (C) C_3.

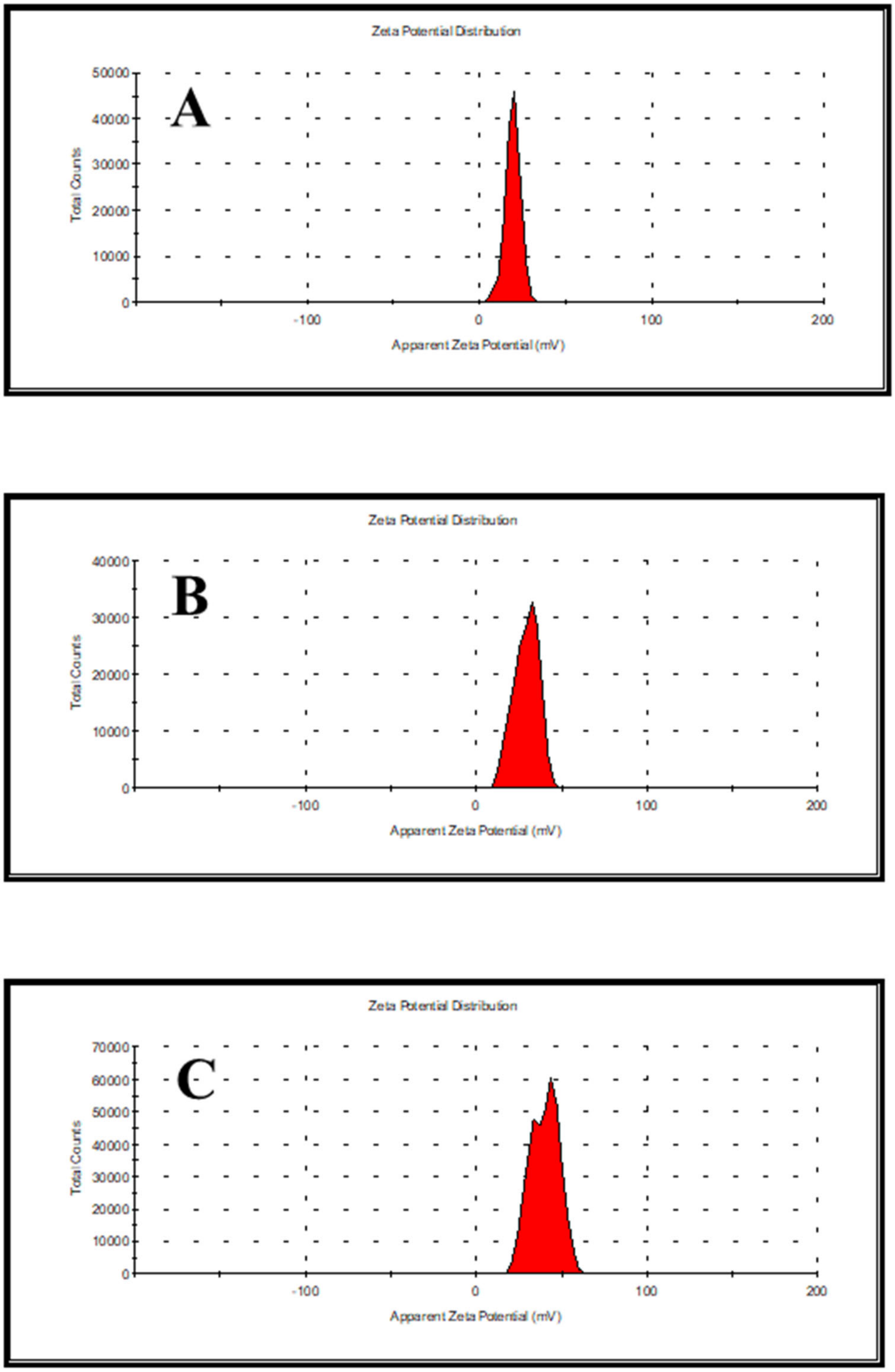

Figure S7. Zeta potential of Au@16-Ph-16 nanoparticles at different $C_{Au@16\text{-}Ph\text{-}16}$ concentrations in PBS 0.1x (ionic strength = 1.63 mM, PH = 7.4). (A) N_1, (B) N_2 and (C) N_3.

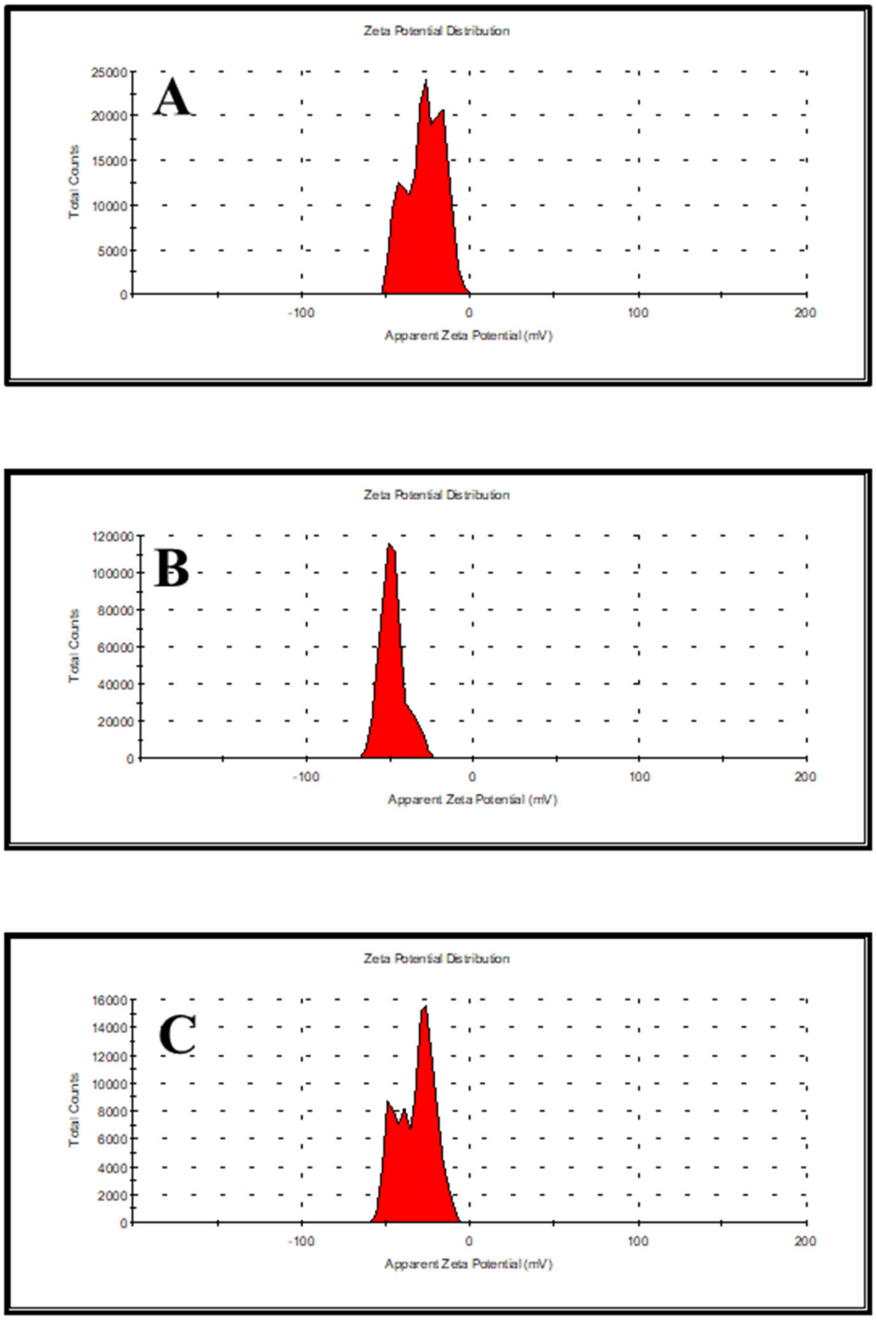

Figure S8. Zeta potential of Au@16-Ph-16/DNA-Doxo compacted nanocomplexes for different formulations in PBS 0.1x (ionic strength = 1.63 mM, PH = 7.4). (A) C_1, (B) C_2 and (C) C_3.

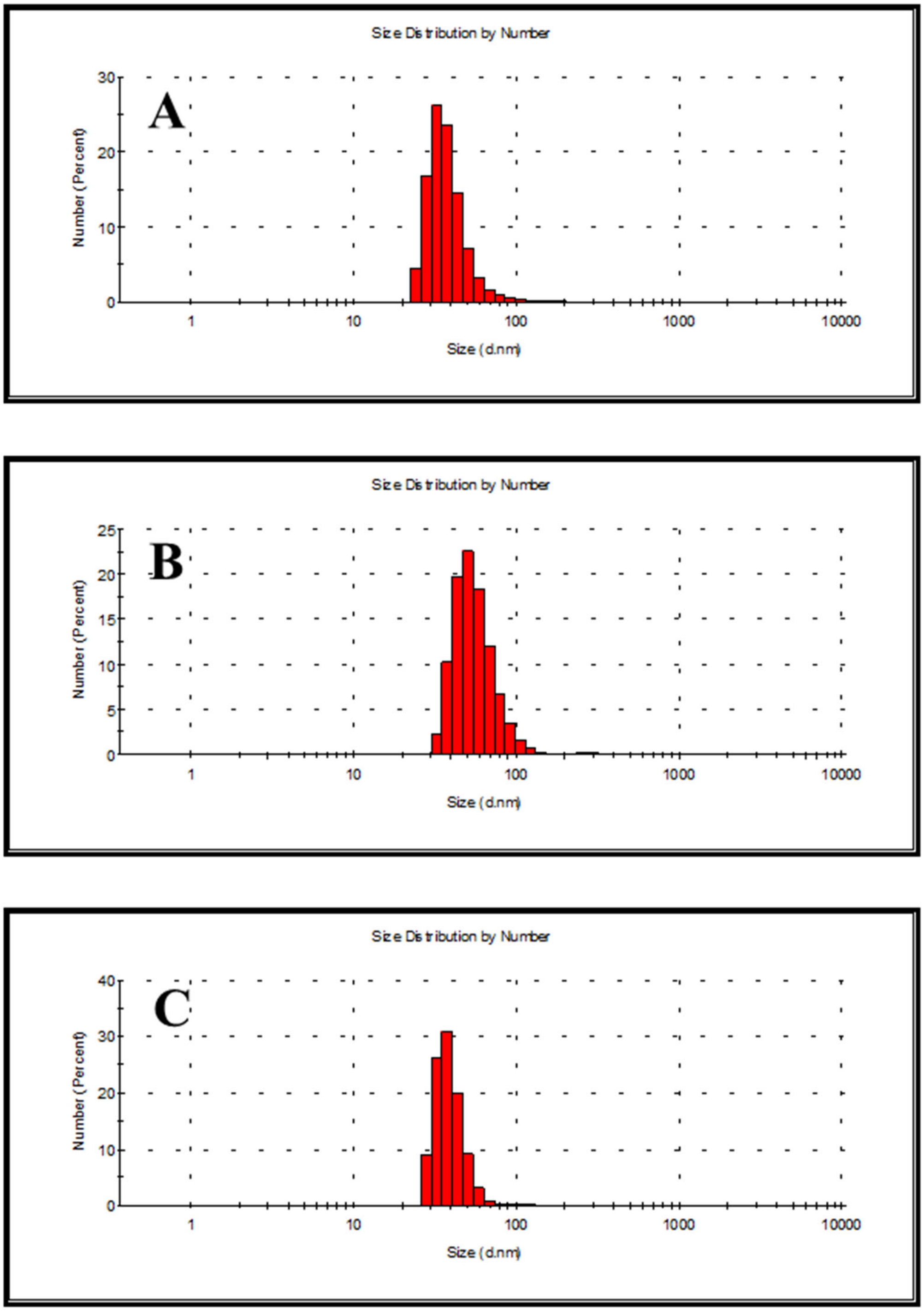

Figure S9. DLS size distribution by number of Au@16-Ph-16/DNA-Doxo compacted nanocomplexes for different formulations in water. (A) C_1, (B) C_2 and (C) C_3.

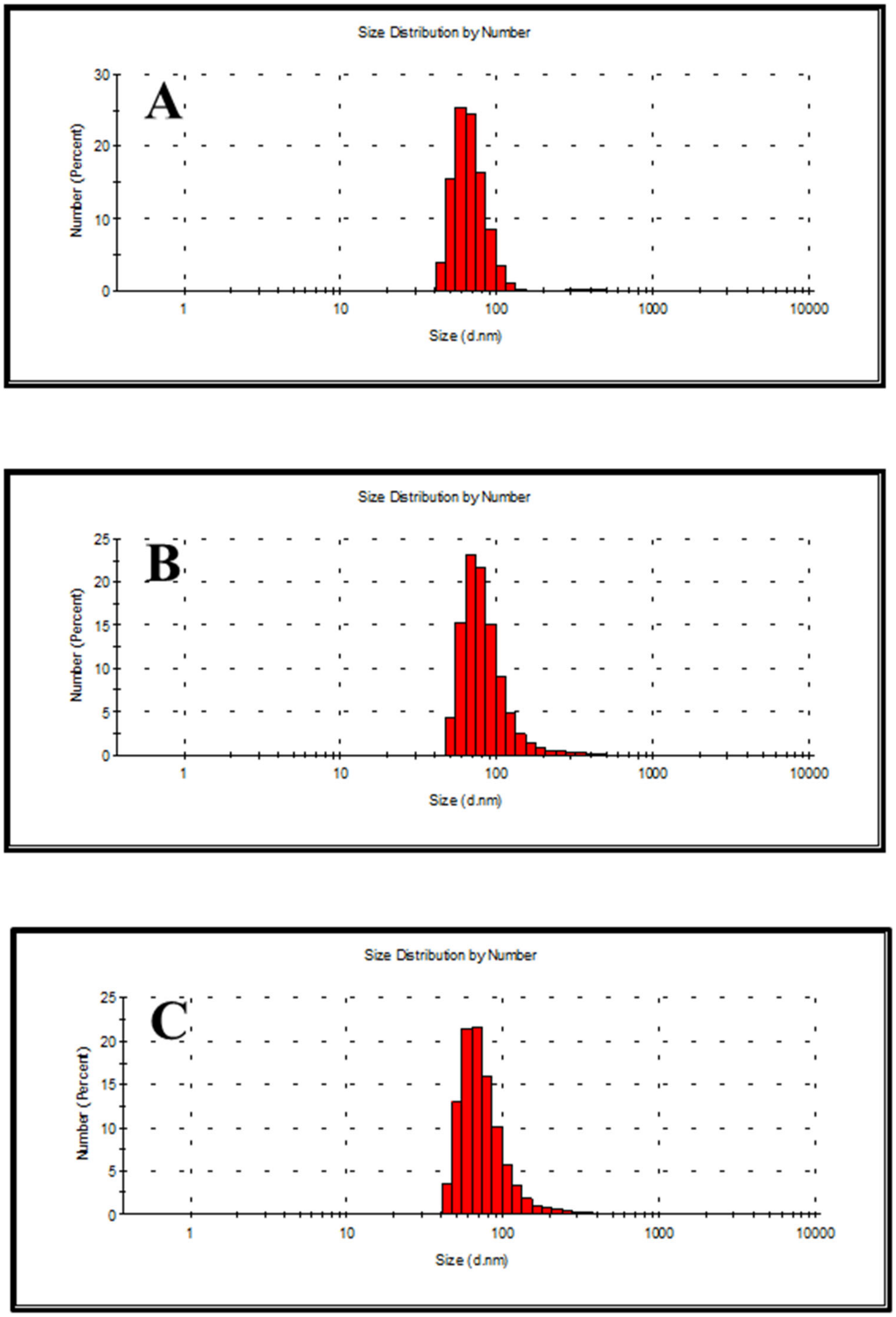

Figure S10. DLS size distribution by number of Au@16-Ph-16/DNA-Doxo compacted nanocomplexes for different formulations in PBS 0.1x (ionic strength = 1.63 mM, PH = 7.4). (A) C_1, (B) C_2 and (C) C_3.

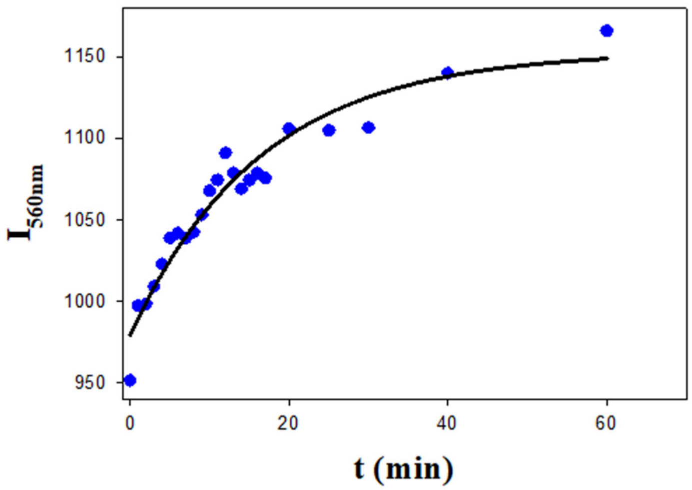

Figure S11. Intro release profile of Doxo from C_3 compacted nanosystems upon addition of N_3 precursor.

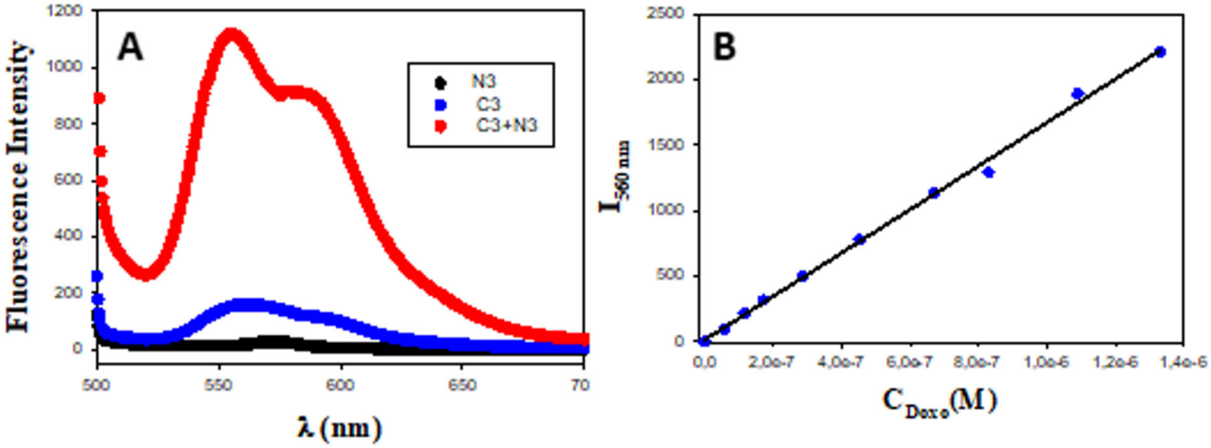

Figure S12. Release assay for C_3 nanocomplex. The spectra of C_3 system is displayed immediately before mixing (in blue) and after mixing with N_3, stabilization and complete Doxo release (in red). The fluorescence spectra recorded on the solution of free N3 (in black) was measured for comparative purpose. All the formulations were prepared as described in section 2.1.4 of the paper. (A) C_3 release in the presence of N_3 nanoparticles; (B) Doxo calibration curve used for measuring released-drug in the presence of N_3.

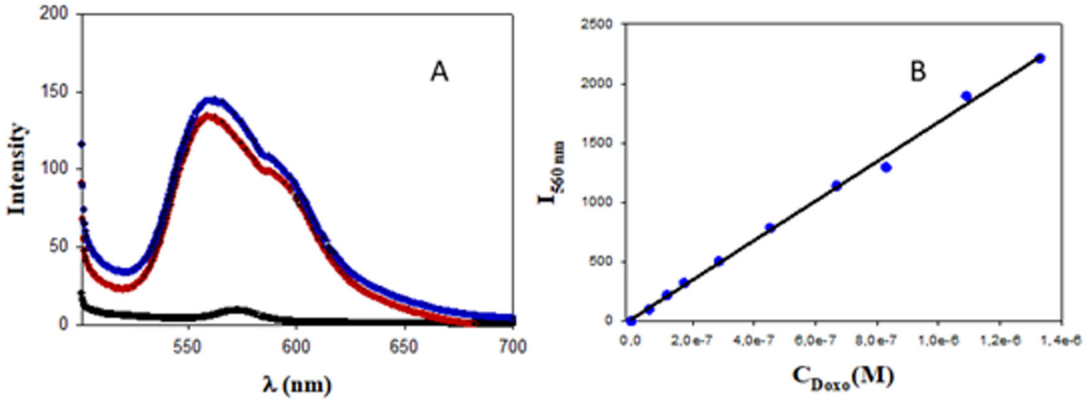

Figure S13. Loading assay for C_3 nanocomplex. The spectra of C_3 system is displayed upon 48 hours of stabilization (in blue) and after correction with Au@16-Ph-16 contribution (in red). Spectra in black corresponds to Au@16-Ph-16 concentration used to prepare the C_3 nanocomplex. C_3 formulation was prepared as described in section 2.1.4 of the paper. (A) C_3 loading; (B) Doxo calibration curve used for measuring encapsulation efficiency.

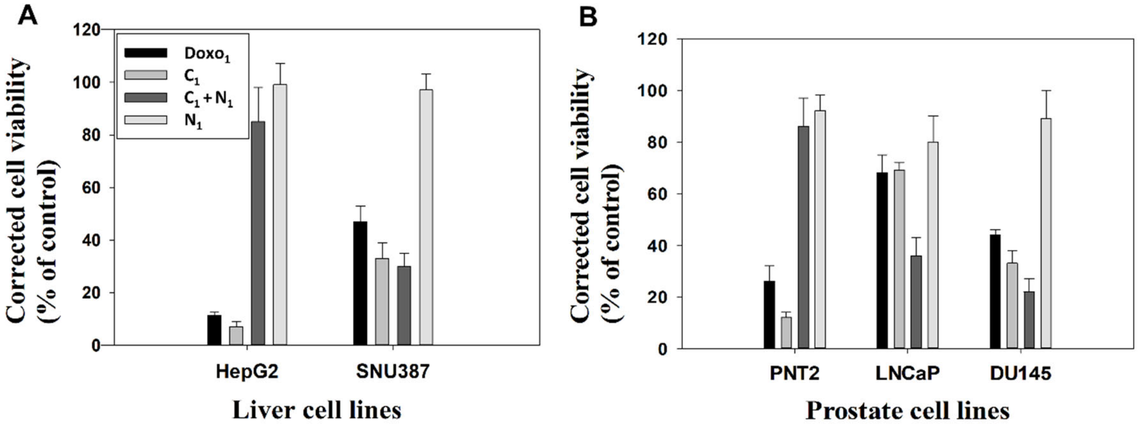

Figure S14. Corrected Cell Viability of treated cells versus control at 48 hours after treatment with $Doxo_1$, C_1, C_1+N_1 and N_1 formulations. Viability of the control corresponds to 100%. (A) HepG2 and SNU387 liver cell lines. (B) PNT2, LNCap and DU145 cell lines.

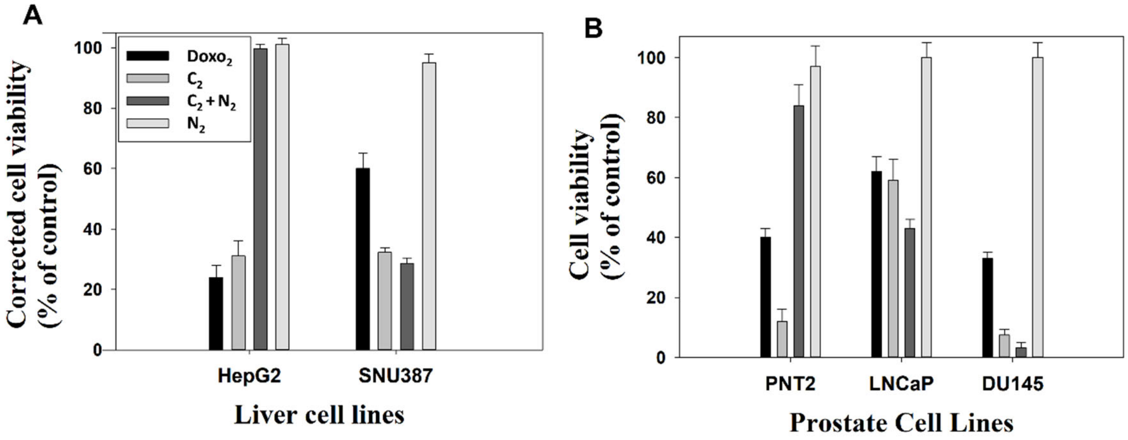

Figure S15. Corrected Cell Viability of treated cells versus control at 48 hours after treatment with $Doxo_2$, C_2, C_2+N_2 and N_2 formulations. Viability of the control corresponds to 100%. (A) HepG2 and SNU387 liver cell lines. (B) PNT2, LNCap and DU145 cell lines.

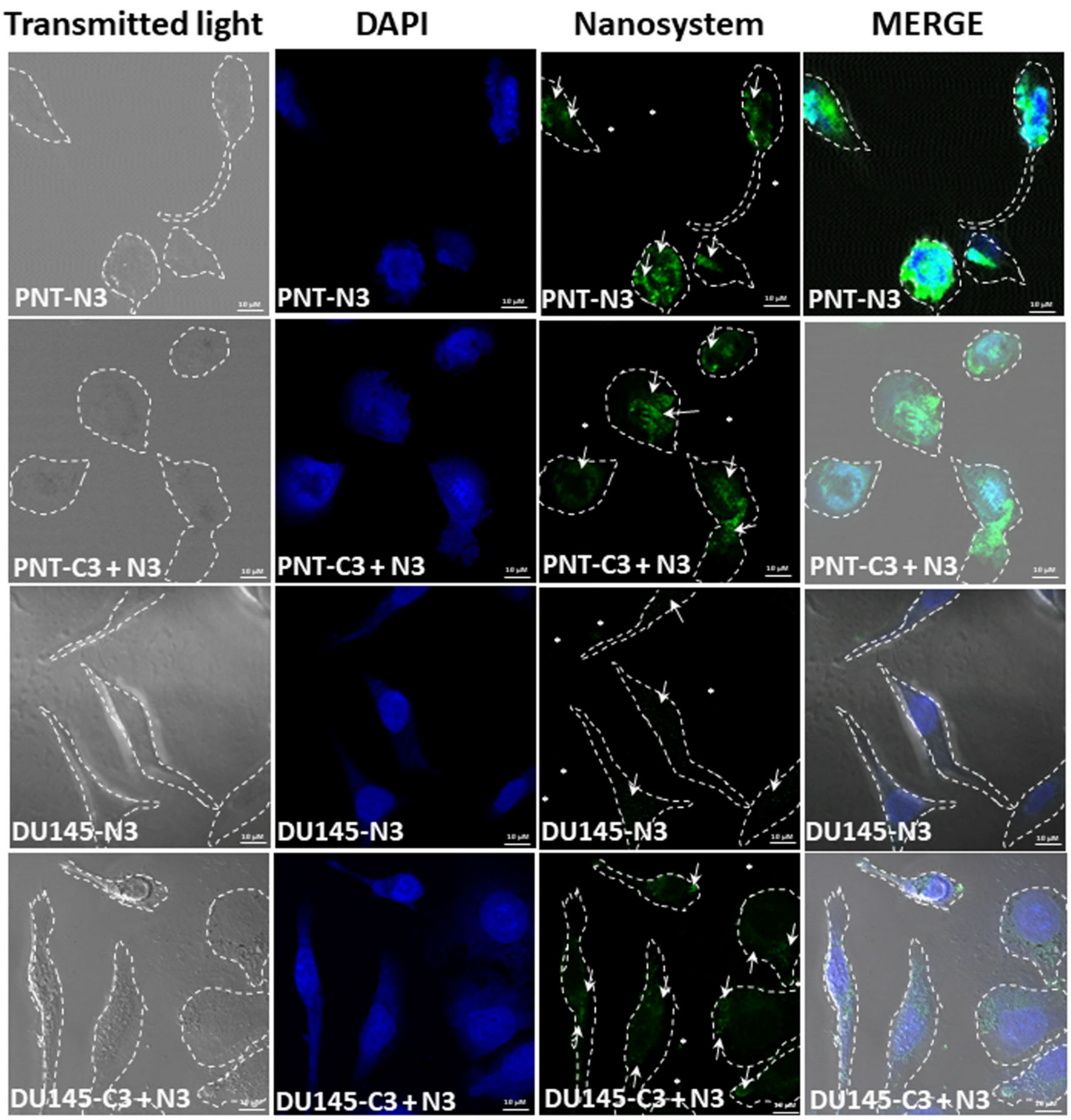

Figure S16. Confocal microphotographs showing cells treated for 24 hours with Au@16-Ph-16 (N3) and Au@16-Ph-16/DNA/Doxo + Au@16-Ph-16 (C3 + N3), in non-tumorigenic prostate PNT2 cells considered control cells and DU145 prostate tumor-derived tumor cells, a castration-resistant model of prostate cancer that does not respond to hormone therapy. First column shows transmitted light microphotography, second column DAPI-labeled nuclei, third column green fluorescently labeled Nanoparticles and Nanosystems, and fourth column merge. The presence of nanoparticles is evidenced by green fluorescence acquired with the 63x/oil objective. Dashed lines mark the contours of cells taken from bright field

images. Arrows indicate nanoparticles inside the cell and asterisks indicate **nanoparticles outside the cell.**

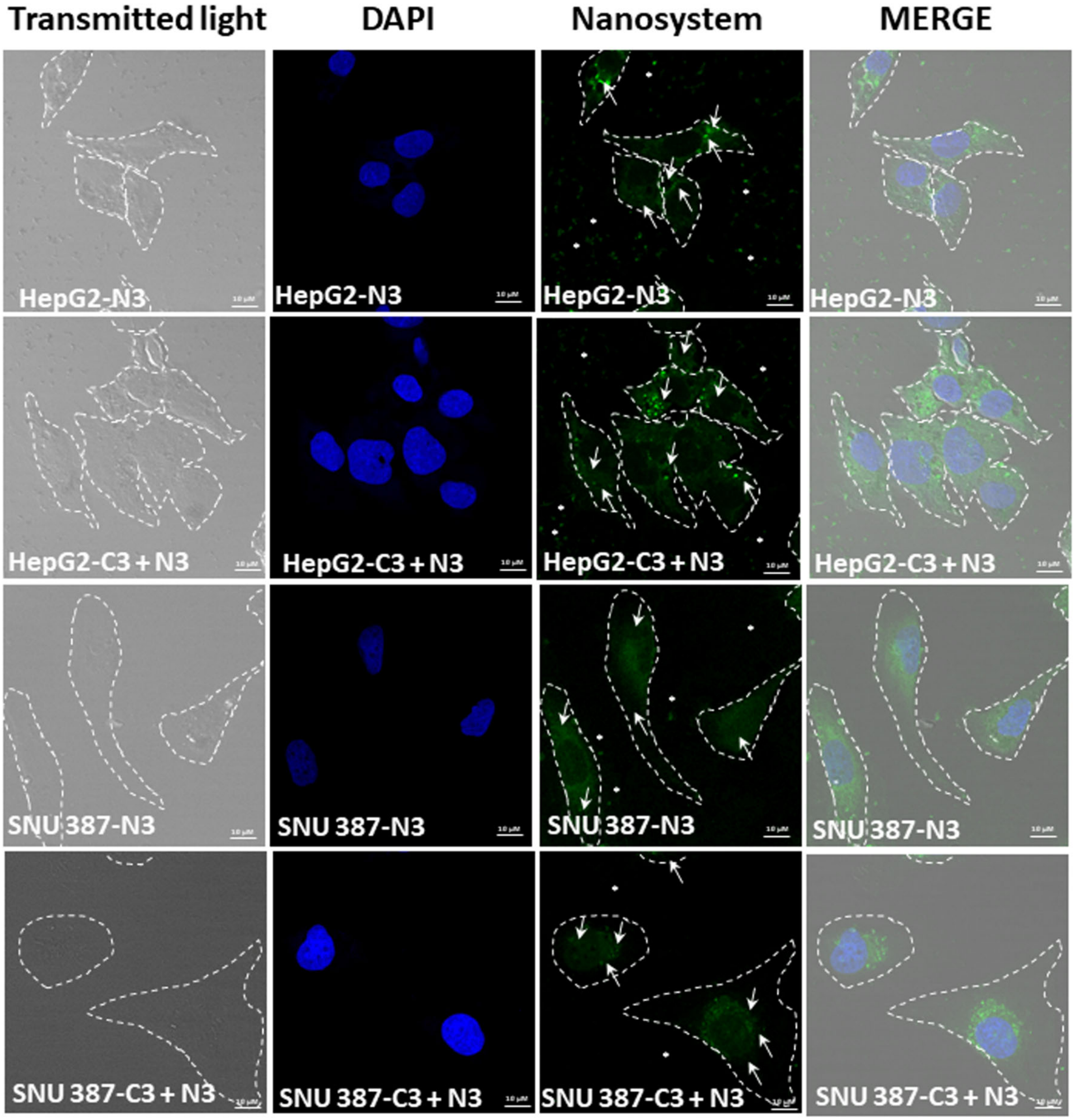

Figure S17. Confocal microphotographs showing cells treated for 24 hours with Au@16-Ph-16 (N3) and Au@16-Ph-16/DNA/Doxo + Au@16-Ph-16 (C3 + N3), in HepG2 cells, derived from non-tumorigenic hepatoblastoma, considered control cells and SNU387 cells, derived from liver tumor, a model of high-grade hepatocarcinoma. First column shows transmitted light microphotography, second column DAPI-labeled nuclei, third column green fluorescently labeled Nanoparticles and Nanosystems, and fourth column merge. The presence of nanoparticles is evidenced by green fluorescence acquired with the 63x/oil objective.

Dashed lines mark the contours of cells taken from bright field images. Arrows indicate nanoparticles inside the cell and asterisks indicate nanoparticles outside the cell.

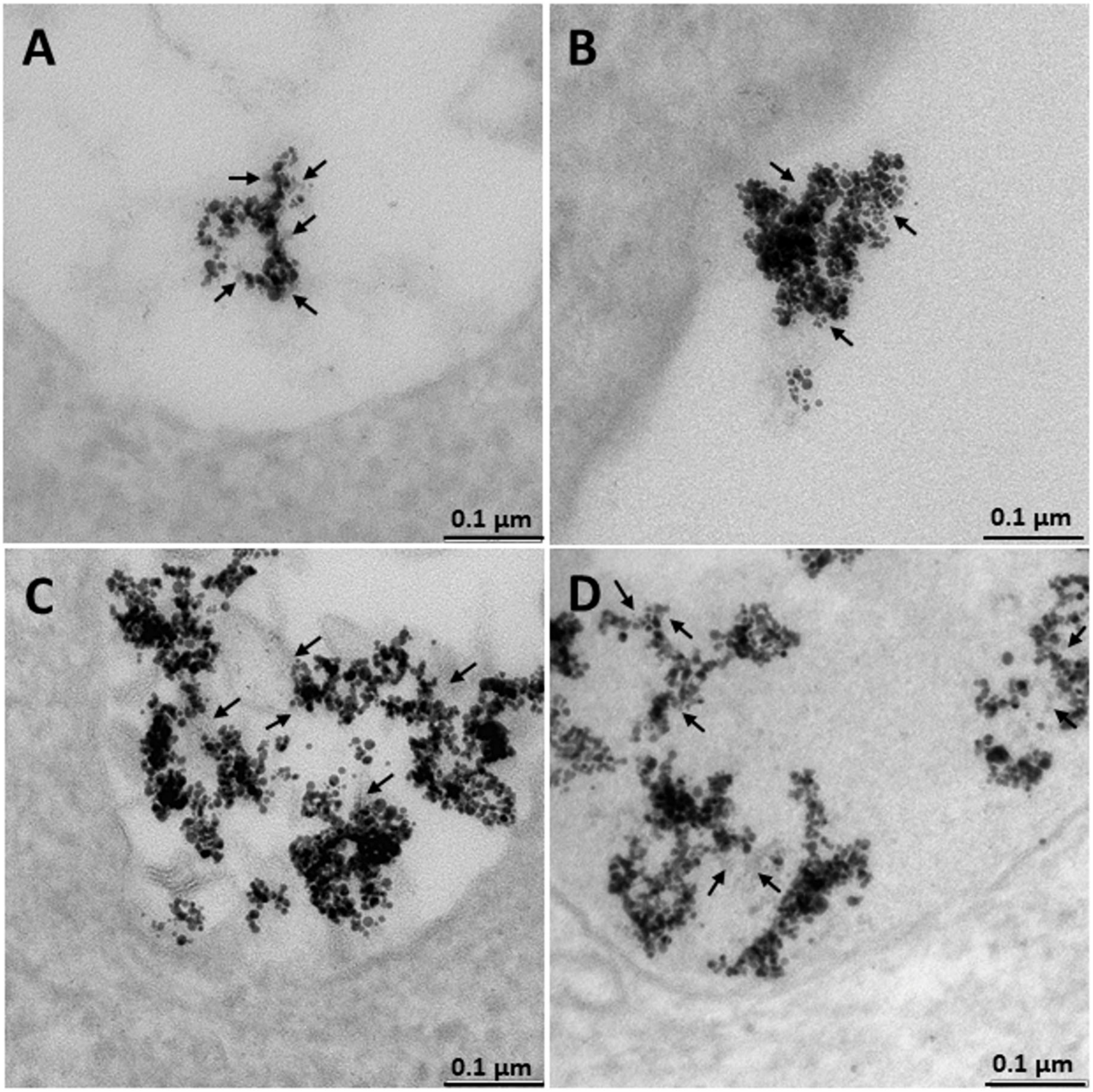

Figure S18. Transmission electron micrograph (TEM) photomicrographs of different tumorigenic and non-tumorigenic cells treated with Au@16-Ph-16/DNA-Doxo (C_3) and Au@16-Ph-16 (N_3). (A) DU145 treated with C_3. (B) PNT2 treated with C_3. (C) HePG2 treated with C_3+ N_3 (D) SNU387 treated with C_3+N_3. The dense bodies correspond to the gold cores of nanoparticles and an arrow in the photograph indicates the DNA biopolymer.

References

[1] Karukstis, K.K.; Thompson, E.H.Z.; Whiles, J.A.; Rosenfeld, R. J. Deciphering the fluorescence signature of daunomycin and doxorubicin. *Biophys. Chem.* **1998**, 73, 249-263. https://doi.org/10.1016/S0301-4622(98)00150-1.

[2] Grueso, E.; Roldan, E.; Perez-Tejeda, P.; Kuliszewska, E.; Molero, B.; Brecker, L.; Giráldez-Pérez, R.M. Reversible DNA Compaction Induced by Partial Intercalation of 16-Ph-16 Gemini Surfactants: Evidence of Triple Helix Formation. *Phys. Chem. Chem. Phys.* **2018**, *20*, 24902–24914. https://doi.org/10.1039/C8CP02791A

[3] Giráldez-Pérez, R.M.; Grueso, E.; Domínguez, I.; Pastor, N.; Kuliszewska, E.; Prado-Gotor, R.; Requena-Domenech, F. Biocompatible DNA/5-Fluorouracil-Gemini Surfactant-Functionalized Gold Nanoparticles as Promising Vectors in Lung Cancer Therapy. *Pharmaceutics* **2021**, *13*, 423. https://doi.org/10.3390/pharmaceutics13030423
